# Aldolase a in pan-cancer and lung squamous cell carcinoma: prognostic value and macrophage-driven immune suppression unveiled by multi-omics and cohort validation

**DOI:** 10.1186/s12935-025-04043-y

**Published:** 2025-11-14

**Authors:** Ying Ji, Xincheng Li, Xihao Shen, Xiumei Hu, Yujing Du, Bin Hu, Wei Wang, Fanjie Meng

**Affiliations:** 1 Department of Thoracic Surgery, Beijing Institute of Respiratory Medicine and Beijing Chao-Yang Hospital, Capital Medical University, NO.8 Gongti South Road, Beijing, 100020 Chaoyang District China; 2 Beijing Chao-Yang Hospital, Capital Medical University, NO.8 Gongti South Road, Beijing, 100020 Chaoyang District China; 3 Department of Pathology, Beijing Chaoyang Hospital, Capital Medical University, Beijing, 100020 China; 4 Department of Nuclear Medicine, Beijing Chaoyang Hospital, Capital Medical University, Beijing, 100020 China

**Keywords:** Aldolase a (ALDOA), Lung squamous cell carcinoma (LUSC), Tumor-associated macrophages, Immune suppression, Prognostic biomarker

## Abstract

**Background:**

Aldolase A (ALDOA), a key glycolytic enzyme, has been implicated in tumor progression and metabolic reprogramming across multiple cancers [1]. However, its role in lung squamous cell carcinoma (LUSC) remains largely unexplored. Recent studies suggest that ALDOA may influence the tumor immune microenvironment, particularly through its association with macrophages [2, 3]. This study aims to investigate the prognostic value of ALDOA in LUSC and its role in macrophage-mediated immune suppression.

**Methods:**

We conducted a comprehensive pan-cancer analysis to evaluate ALDOA expression, genomic alterations, and prognostic relevance across different cancer types. In LUSC, we validated its prognostic value using immunohistochemical (IHC) staining and independent patient cohorts. Immune infiltration was assessed using multiple bioinformatics algorithms and single-cell RNA sequencing (scRNA-seq) from the TISCH2 database. Spatial transcriptomics and immunofluorescence (IF) staining were performed to determine ALDOA’s co-localization with CD68 + macrophages in LUSC tissues. Functional enrichment and drug sensitivity analyses were conducted to explore ALDOA’s role in tumor progression and therapeutic resistance.

**Results:**

ALDOA was significantly overexpressed in multiple cancers, with LUSC showing one of the highest expression levels. Elevated ALDOA expression was strongly correlated with poor overall survival (OS), disease-specific survival (DSS), and progression-free interval (PFI) in LUSC patients. Copy number variations and promoter hypomethylation were identified as potential mechanisms driving ALDOA overexpression. ALDOA-high tumors exhibited increased M0 macrophage and reduced CD8 + T-cell infiltration, suggesting a role in immune suppression and evasion. Spatial transcriptomic and immunofluorescence analyses confirmed the co-localization of ALDOA with CD68 + tumor-associated macrophages (TAMs). Additionally, ALDOA-high tumors demonstrated increased resistance to multiple chemotherapeutic agents and EGFR-TKIs, highlighting its potential as a predictive biomarker for drug response.

**Conclusion:**

Our findings establish ALDOA as a robust prognostic biomarker and a key regulator of macrophage-mediated immune suppression in LUSC. Its involvement in tumor metabolism, immune evasion, and therapy resistance suggests that targeting ALDOA could enhance both metabolic inhibition strategies and immune checkpoint blockade therapies. Future research should focus on mechanistic studies and therapeutic interventions targeting ALDOA to improve treatment outcomes in LUSC.

**Supplementary Information:**

The online version contains supplementary material available at 10.1186/s12935-025-04043-y.

## Introduction

Aldolase A (ALDOA) is a crucial glycolytic enzyme that catalyzes the reversible cleavage of fructose-1,6-bisphosphate into glyceraldehyde-3-phosphate and dihydroxyacetone phosphate, playing a fundamental role in cellular energy metabolism [3, 4]. It is widely expressed in various tissues, with particularly high levels in muscle and brain, where it contributes to ATP production under both aerobic and anaerobic conditions [1]. Beyond its metabolic function, ALDOA also regulates cytoskeletal organization, vesicle trafficking, and cell motility [4]. Recent studies have revealed that ALDOA also participates in stress responses and cellular signaling pathways, underscoring its broader biological significance [1].

Accumulating evidence indicates that ALDOA is aberrantly expressed in various malignancies. It also plays a pivotal role in cancer progression [4]. Elevated ALDOA expression has been reported in hepatocellular carcinoma, breast cancer, colorectal cancer, and lung cancer, where it promotes tumor cell proliferation, migration, and invasion [5–8]. Mechanistically, ALDOA has been implicated in metabolic reprogramming, facilitating the Warburg effect by enhancing glycolysis to meet the bioenergetic and biosynthetic demands of rapidly proliferating cancer cells [9]. Additionally, ALDOA interacts with key oncogenic pathways, including the PI3K/AKT/mTOR and Wnt signaling cascades, further driving tumor aggressiveness [10, 11]. Beyond its intrinsic role in tumor cells, ALDOA has also been linked to the tumor microenvironment, influencing immune cell infiltration and immune evasion [12]. Given its broad oncogenic functions, ALDOA has emerged as a potential prognostic biomarker and therapeutic target in multiple cancer types [4]. However, its precise role in lung squamous cell carcinoma remains largely unexplored.

Lung squamous cell carcinoma (LUSC) accounts for approximately 30% of non-small cell lung cancers (NSCLC) and is associated with high morbidity and mortality worldwide [13]. Despite advancements in therapeutic strategies, the prognosis of LUSC remains unsatisfactory, largely due to its aggressive nature and the lack of effective targeted therapies [14, 15]. Identifying reliable prognostic biomarkers is crucial for improving risk stratification and guiding personalized treatment approaches [16]. While ALDOA has been recognized as a potential oncogenic driver in several malignancies, its specific role in LUSC and the underlying molecular mechanisms remain poorly understood. In particular, its interaction with the tumor immune microenvironment and its contribution to immune evasion require further investigation.

The pan-cancer approach—integrating multi-omics data across diverse cancer types—has emerged as a powerful strategy in oncology research. This methodology offers a broader, more systematic perspective that can uncover common molecular mechanisms and universal oncogenic drivers transcending tissue-of-origin limitations. By identifying consistently altered pathways and biomarkers across cancers, pan-cancer studies can prioritize robust therapeutic targets and prognostic indicators with wider clinical applicability. The value of this approach has been successfully demonstrated in recent studies elucidating the roles of various molecules, such as PLIN3 in lipid metabolism and macrophage infiltration [17], EPHB2 as a novel predictor for immunotherapy [18], the interplay between disulfidptosis and the tumor microenvironment [19], and the immunotherapeutic potential of SLC35A2 [20]. Inspired by these findings, our study adopts a pan-cancer framework to first establish the overarching significance of ALDOA, followed by a deep dive into its specific role and mechanism in LUSC, thereby aiming to delineate both its universal and context-dependent oncogenic functions.

In this study, we conducted a comprehensive pan-cancer analysis of ALDOA, with a particular focus on its expression patterns, prognostic significance, and genomic alterations in LUSC. We further validated its prognostic value in independent LUSC cohorts and explored its association with immune infiltration, particularly its macrophage-mediated effects. Additionally, immunofluorescence staining was performed to confirm the co-localization of ALDOA with CD68-positive macrophages in LUSC tissues. Our findings provide novel insights into the oncogenic role of ALDOA in LUSC and highlight its potential as a prognostic biomarker and therapeutic target.

## Methods

### Pan-cancer data collection and processing

Pan-cancer data, including TCGA tumor samples and GTEx normal tissue samples, were retrieved from the USUC Xena platform (https://xena.ucsc.edu/) to evaluate the expression of ALDOA across various cancer types. The expression levels of ALDOA were compared between tumor and normal tissues. Protein expression data for ALDOA were analyzed using the Clinical Proteomic Tumor Analysis Consortium (CPTAC) dataset, available through the UALCAN portal (https://ualcan.path.uab.edu/analysis-prot.html). Immunohistochemical staining for ALDOA in lung squamous cell carcinoma (LUSC) was obtained from the Human Protein Atlas (HPA, https://www.proteinatlas.org/). Additionally, microarray data for LUSC patients from the GSE37734 dataset were extracted for survival analysis (https://www.ncbi.nlm.nih.gov/geo/query/acc.cgi? acc=GSE37734).

### ALDOA diagnostic and prognostic analysis

Receiver operating characteristic (ROC) curves for cancers of interest were generated using the R ‘pROC’ package to assess the diagnostic performance of ALDOA expression. Survival analysis was performed to compare the outcomes of individuals with high and low ALDOA expression levels using Kaplan-Meier curves, with patients stratified according to the median ALDOA expression. The R ‘survival’ and ‘survminer’ packages were used to generate survival curves. To evaluate the prognostic significance of ALDOA expression, univariate Cox regression analyses were conducted using the R ‘survival’ and ‘forestplot’ packages, assessing overall survival (OS), disease-specific survival (DSS), and progression-free interval (PFI).

### Genomic alteration and mutational burden analyses

Pan-cancer genomic alterations, including mutations, amplifications, and deletions, were analyzed using the cBioPortal Cancer Type Summary module. Tumor mutational burden (TMB) and microsatellite instability (MSI) were evaluated using the R ‘maftools’ package^15^. In addition, analyses of Mutant-Allele Tumor Heterogeneity (MATH), ploidy, loss of heterozygosity (LOH), and homologous recombination deficiency (HRD) were conducted using the Sangerbox platform. Kaplan-Meier survival curves for ALDOA copy number variations (CNVs) were obtained from the UALCAN database to evaluate their prognostic significance.

### Methylation and epigenetic modification analysis

ALDOA promoter methylation was analyzed using the UALCAN database, with additional methylation data for LUSC retrieved from the MEXPRESS platform (https://mexpress.ugent.be/). The TIDE methylation module was used for pan-cancer Kaplan-Meier survival analysis. The relationship between PSME2 and the expression of N1-methyladenosine (m1A), 5-methylcytosine (m5C), and N6-methyladenosine (m6A)-modifying genes was assessed using heatmaps.

### ALDOA alternative splicing analyses

Clinically relevant alternative splicing events of ALDOA were investigated using the OncoSplicing server with the ClinicalAS tool (https://www.cbioportal.org/). Relevant ALDOA splicing events were identified in the SpliceSeq and SplAdder projects. Percent spliced-in (PSI) data for TCGA cancer and GTEx tissue samples were analyzed for survival differences, visualized using PanPlot and Bubbleplot.

### Functional enrichment analysis

Gene Set Variation Analysis (GSVA) was performed using the “GSVA” R package to explore the biological characteristics of each cluster [21]. HALLMARK and KEGG gene sets from the MSigDB were incorporated into the analysis. Enrichment scores were calculated using the “limma” package for differential analysis. Additionally, GSEA was performed for ALDOA based on its correlation with other genes.

### Comprehensive tumor microenvironment (TME) analysis

Immune infiltration in tumor samples was assessed using various immune cell composition algorithms from the IOBR package [22]. The ssGSEA algorithm quantified immune cell infiltration and analyzed immune-related pathway activity. To evaluate immune escape potential and predict response to immunotherapy, the tumor immune dysfunction and rejection (TIDE) score was calculated. Immunophenoscore (IPS) data from the Cancer Immunome Atlas (https://tcia.at/home) were used to identify LUSC patients likely to benefit from immunotherapy. Immune checkpoint genes were curated from the literature. Tumor mutational burden (TMB) for each patient was calculated using TCGA somatic mutation data with the R package “maftools.” Drug sensitivity to chemotherapeutic agents was assessed by estimating IC50 values through the “oncoPredict” package and the Genomics of Drug Sensitivity in Cancer database [23]. Ridge regression models were employed to predict individual drug responses.

### Transcriptome analysis at the single-cell and Spatial level

Pan-cancer single-cell RNA-seq datasets were retrieved from the Tumor Immune Single-cell Hub 2 (TISCH2, http://tisch.comp-genomics.org/) platform to analyze ALDOA expression in different cell types [24]. Spatial transcriptomic data from the STOmicsDB platform were used to assess the co-expression of ALDOA and CD68 [25].

### Immunotherapy cohort and biomarker analysis

To independently validate the predictive value of ALDOA for response to immune checkpoint blockade (ICB), we utilized the TIGER database (http://tiger.canceromics.org/). This comprehensive resource integrates bulk transcriptomic data from 1,508 tumor samples with annotated clinical immunotherapy outcomes (spanning 8 cancer types) and 11,057 samples without immunotherapy information (33 cancer types), alongside single-cell RNA sequencing data from 2,116,945 immune cells across 655 samples (25 cancer types). Additionally, TIGER collates 31 published CRISPR screening datasets focused on tumor-immune interactions.

Within the TIGER platform, we specifically employed the “Immunotherapy Response” module. ALDOA expression was compared between the Responder group (defined as patients achieving a complete or partial response, CR/PR) and the Non-Responder group (defined as patients with stable or progressive disease, SD/PD) in the non-small cell lung cancer (NSCLC) cohort from the GSE135222 dataset, which is included within TIGER. Furthermore, the predictive performance of ALDOA for immunotherapy response was evaluated by calculating the Area Under the Curve (AUC) value. Its performance was systematically compared against several established immune response signatures, including the T cell-inflamed gene expression profile (GEP), cancer-associated fibroblast (CAF), M2 tumor-associated macrophages (TAM M2), IFNG, CD8, CD274 (PD-L1), tertiary lymphoid structures (TLS), and a melanoma-specific TLS signature.

### Analysis of ALDOA expression in normal lung at single-cell resolution

To delineate the cellular origin of ALDOA expression in the lung microenvironment and to validate its specific enrichment in macrophages, we interrogated the Human Protein Atlas (HPA) database (https://www.proteinatlas.org/). Using the “Cell Type Enrichment” tool within the “Single Cell Type” section, we retrieved single-cell RNA sequencing data for the ALDOA gene in normal human lung tissue. The analysis included: A UMAP plot and a corresponding bar chart visualizing ALDOA RNA expression across all identified cell clusters in the lung. Two cell type marker heatmaps (from the “Single Cell Type” and “Tabula Sapiens” projects) displaying the expression of ALDOA alongside canonical macrophage markers (CD163, CD68, MARCO, MRC1, MSR1). Expression values were normalized as Z-scores to facilitate cross-gene comparison.

### Cells and reagents

The murine lung squamous carcinoma cell line KLN 205 was obtained from the Institute of Basic Medical Sciences of the Chinese Academy of Medical Sciences. KLN 205 cells were cultured in high-glucose DMEM (Gibco, USA) supplemented with 10% fetal bovine serum (Gibco, USA) at 37 °C under a 5% CO2 atmosphere.

### Tumor and tissue collection in KLN 205 allograft model

Eight-week-old female C57BL/6J mice were purchased from Beijing HFK Bioscience Co., LTD (Beijing, China) and maintained in a standard SPF laboratory environment (specific pathogen-free, temperature-controlled conditions with a 12 h light/dark cycle). After one week of environmental acclimatization, KLN 205 cells (2 × 106 cells) were orthotopically implanted into the left lung of mice in a 0.1 mL suspension. Ten tumor-bearing mice were euthanized for sample collection, with three tissue types harvested from each animal: primary tumor (Tumor), adjacent normal lung tissue (Adj), and regional lymph nodes (NL). All collected tissues were rinsed three times with prechilled phosphate-buffered saline (PBS), flash-frozen in liquid nitrogen, and stored at −80 °C for subsequent analysis. This study was approved by the Animal Experimental Ethics Committee of Capital Medical University (Approval No.: AEEI-2024-357), with all experimental procedures strictly adhering to institutional guidelines for the humane care and use of laboratory animals.

### Anti-PD-1 treatment in the KLN 205 allograft model

To evaluate the therapeutic response and its correlation with Aldoa expression, a separate cohort of KLN 205 tumor-bearing mice was established as described above. Once the tumor volume reached approximately 100 mm³, the mice were randomly divided into two groups (*n* = 3–4 per group): the control group and the anti-PD-1 treatment group. Mice in the treatment group received intraperitoneal injections of anti-PD-1 antibody (clone RMP1-14, Bio X Cell) at a dose of 200 µg per mouse every three days for a total of 17 days. The control group received isotype control antibody (rat IgG2a, Bio X Cell) on the same schedule. Tumor volumes were measured and calculated using a digital caliper every three days with the formula: Volume = (Length × Width²)/2. After 17 days of treatment, all mice were euthanized, and tumor tissues were harvested. The tumor was snap-frozen in liquid nitrogen for subsequent proteomic analysis (Aldoa measurement).

### Extraction and digestion of proteins

Tissues were homogenized in lysis buffer consisting of 1% sodium deoxycholate, 10 mM Tris (2-carboxyethyl) phosphine, 40 mM 2-chloroacetamide and 100 mM Tris-HCl pH8.8. After heating at 95 °C for 5 min and sonicating for 5 min (3 s on and 3 s off, amplitude 25%), the tissue lysates were centrifuged at 16,000 g for 10 min at 4 °C, and the supernatants were collected as whole tissue extract (WTE). Their protein concentration was determined by Thermo Nanodrop One (Thermo Fisher, USA). Cell lysates of 100 µg protein were digested overnight with trypsin (Promega, USA) at 37 °C, and the digestion was stopped by formic acid at the final concentration of 1%. Precipitated sodium deoxycholate was removed by centrifugation at 4 °C with 16,000 g for 10 min. The supernatants were collected, desalted, vacuum-dried and stored at −80 °C until subsequent liquid chromatography tandem mass spectrometry liquid chromatography tandem mass spectrometry (LC-MS/MS) analysis.

### LC-MS/MS analysis

MS samples were analyzed on a Orbitrap Fusion mass spectrometer (Thermo Fisher Scientific, Rockford, IL, USA) (Thermo Fisher Scientific) coupled online to an Easy-nLC 1,000 nanoflow LC system (Thermo Fisher Scientific). Vacuum-dried samples were dissolved with 24 ul phase A (0.1% formic acid water), vortexed on the shaker for 15 s, and centrifuged at 14,000 g for 10 min. 1/8 of the supernatant was used for analysis. The sample was separated by a 150 μm × 30 cm silica microcolumn (homemade; particle size, 1.9 μm; pore size, 120 Å) with a linear gradient of 6–40% and 95% Mobile Phase B (0.1% formic acid in acetonitrile) at a flow rate of 600 nl/min for 140 min and 10 min, respectively. To acquire mass spectra, data-dependent mode was applied by carrying out a Full MS scan (AGC target 2 × 105 ions, maximum injection time 50 ms, 300–1400 m/z, *R* = 120,000 at 200 m/z) followed by a duty cycle of 3 s was performed in Rapid mode with high-energy collision dissociation (target 5 × 103 ions, max injection time 35 ms, isolation window 1.6 m/z, normalized collision energy of 35%), detected in the Iontrap (*R* = 15,000 at 200 m/z). Dynamic exclusion time was set to 25 s. All data were acquired using the Xcalibur software (Thermo Fisher Scientific).

### Peptide identification and protein quantification

MS raw files were processed with the Z-system proteomics work-station (http://61.155.143.3:9000/). MS raw files were searched against the National Center for Biotechnology Information (NCBI) Ref-seq mouse proteome database (updated on 04/07/2013, 27,414 entries) in Mascot search engine (version 2.3, Matrix Science Inc.). The mass tolerances were 20 ppm for precursor ions and 50 mmu or 0.5 Da for productions. The trypsin proteolytic cleavage sites are KR. Up to two missed cleavages were allowed. The minimal peptide length was seven amino acid long. Cysteine carbamidomethylation was set as a fixed modification, and protein N-acetylation and oxidation of methionine were considered variable modifications, and the charges of precursor ions were limited to + 2, +3, + 4 and + 5. All identified peptides were quantified in Z-system with peak areas derived from their MS1 intensity. Peptide false discovery rate (FDR) was adjusted to 1%. For protein level, we kept the proteins that had at least one unique peptide and two high-confidence peptides (mascot ion score > 20). Protein quantification was determined by a label-free, intensity-based absolute quantification (iBAQ) approach according to the area under the curve (AUC) of precursor ions which was calculated with homemade software. The fraction of total (FOT) was calculated by this formula: protein’s iBAQ/the total iBAQ. The FOT was further multiplied by 105 to obtain the relative abundance (iFOT) for easy representation. Missing values were substituted with zeros.

### Mouse proteomic analysis

Differential protein expression analysis was conducted using the “limma” method. Gene Ontology (GO) and KEGG pathway analyses were performed with the R packages “clusterProfiler” and “org.Mm.eg.db” [26]. Gene Set Enrichment Analysis (GSEA) was carried out using the “m2.cp.v2024.1.Mm.symbols.gmt” and “m5.go.v2024.1.Mm.symbols.gmt” gene sets downloaded from the MsigDB.

### Patient enrollment and tissue microarray construction

This study employed a retrospective cohort design, systematically screening consecutive cases of patients who underwent radical lobectomy in the Department of Thoracic Surgery of our hospital between January 2011 and December 2020. Inclusion criteria were: (1) postoperative pathological confirmation of lung squamous cell carcinoma; (2) availability of complete R0 resection specimens (microscopically negative margins); (3) complete clinicopathological data and follow-up records. Exclusion criteria included: (1) history of neoadjuvant therapy; (2) intraoperative frozen section indicating positive margins; (3) M1-stage disease according to the 9th edition TNM staging system; (4) paraffin-embedded tissue blocks failing to meet technical standards (e.g., tissue detachment, inadequate fixation). Initial screening identified 170 eligible cases, with 7 specimens excluded during secondary pathological quality control (4 due to fixation-induced shrinkage reducing tumor cell proportion to < 30%, and 3 due to tissue compression artifacts). Ultimately, 163 cases were included for tissue microarray construction.

Tissue microarray (TMA) preparation strictly followed international standardized protocols (TMA Master, 3DHistech). Two senior pathologists independently identified tumor cell-dense regions (tumor cell proportion ≥ 60%) under a multi-head microscope, performing triplicate core sampling using a 1.5 mm tissue puncher. Each TMA contained a 163 core matrix, with 5% control cores (including normal lung tissue and standardized squamous cell carcinoma cell lines) for staining quality control. All sections were prepared as 4 μm continuous slices using a semi-automated microtome. Tissue integrity was verified via H&E counterstaining prior to immunohistochemical and molecular analyses.

### Hematoxylin-eosin (HE) staining

The DIAPATH Donatello dehydrator, Leica RM2016 microtome, and Nikon E100 microscopy system were employed. Key reagents included Servicebio eco-friendly dewaxing solutions, HD hematoxylin staining solution, and eosin staining system. Paraffin sections underwent gradient dewaxing (eco-friendly dewaxing solutions I/II/III, 10 min each) and ethanol gradient rehydration (100%−75%). For frozen sections, after fixation in neutral formalin, sequential staining was performed: hematoxylin immersion (3–5 min, with differentiation solution G1039 to regulate nuclear staining depth and bluing solution to restore physiological pH) and eosin gradient staining (15 s cytoplasmic staining after equilibration in 95% ethanol). Quality control included batch-specific normal lung tissue controls (*n* = 3) to verify staining uniformity and double-blind microscopic evaluation (whole-slide scanning with 10× objective) to ensure tumor cell nuclear content ≥ 30%, excluding slices with shrinkage/crushing artifacts. Hematoxylin reagent efficacy was validated monthly using standard liver tissue sections to ensure batch consistency.

### Immunohistochemistry (ALDOA)

Using rabbit-derived anti-ALDOA monoclonal antibody (abcam, ab252953, 1:100) and HRP-labeled goat anti-rabbit IgG secondary antibody (Servicebio, GB23303), antigen epitopes were exposed via microwave heat retrieval with EDTA buffer (pH 9.0) (medium heat for 8 min, pause for 8 min, medium-low heat for 7 min). Endogenous peroxidase activity was blocked with 3% H₂O₂ for 25 min, and nonspecific binding was blocked with 3% BSA. A streptavidin-HRP three-stage amplification system and DAB chromogenic detection were employed (dynamic monitoring under microscopy, with reference to standard controls to regulate chromogenic intensity). Quantitative analysis utilized double-blind semi-quantitative scoring (staining intensity: 0–3 points; positive cell proportion: 0–4 points; H-score calculated) and NIS-Elements software to measure integrated optical density (IOD). The threshold was set as negative control + 3SD to ensure objective results.

### Fluorescence homologous dual labeling staining (ALDOA/CD68)

The fluorescence homologous dual labeling staining (ALDOA/CD68) protocol was performed using Tyramide Signal Amplification (TSA) technology, enabling sequential detection of ALDOA (TSA-CY3, 555 nm) and CD68 (TSA-iF488, 488 nm) through HRP-mediated covalent tyramide deposition. Rabbit anti-ALDOA (1:500) and rabbit anti-CD68 (1:2000) antibodies were sequentially incubated with microwave-mediated antigen stripping (95 °C EDTA buffer, 15 min) to eliminate cross-reactivity. The workflow involved an initial ALDOA detection cycle: overnight primary antibody incubation at 4 °C, HRP-conjugated secondary antibody (50 min), and TSA-CY3 deposition. Following microwave dissociation of immune complexes, the CD68 detection cycle replicated the process with TSA-iF488 deposition. Autofluorescence was minimized using quenching agent G1221, and nuclei were counterstained with DAPI. Whole-slide imaging was conducted on a Pannoramic MIDI system with a 20× objective and Z-stack acquisition (1 μm slice thickness). Manders’ colocalization coefficients were calculated using Definiens Developer XD software, with single-label calibration ensuring ≤ 5% spectral crosstalk between channels.

## Results

### Expression levels and clinical relevance of ALDOA in pan-cancer and LUSC

**Fig. 1:**
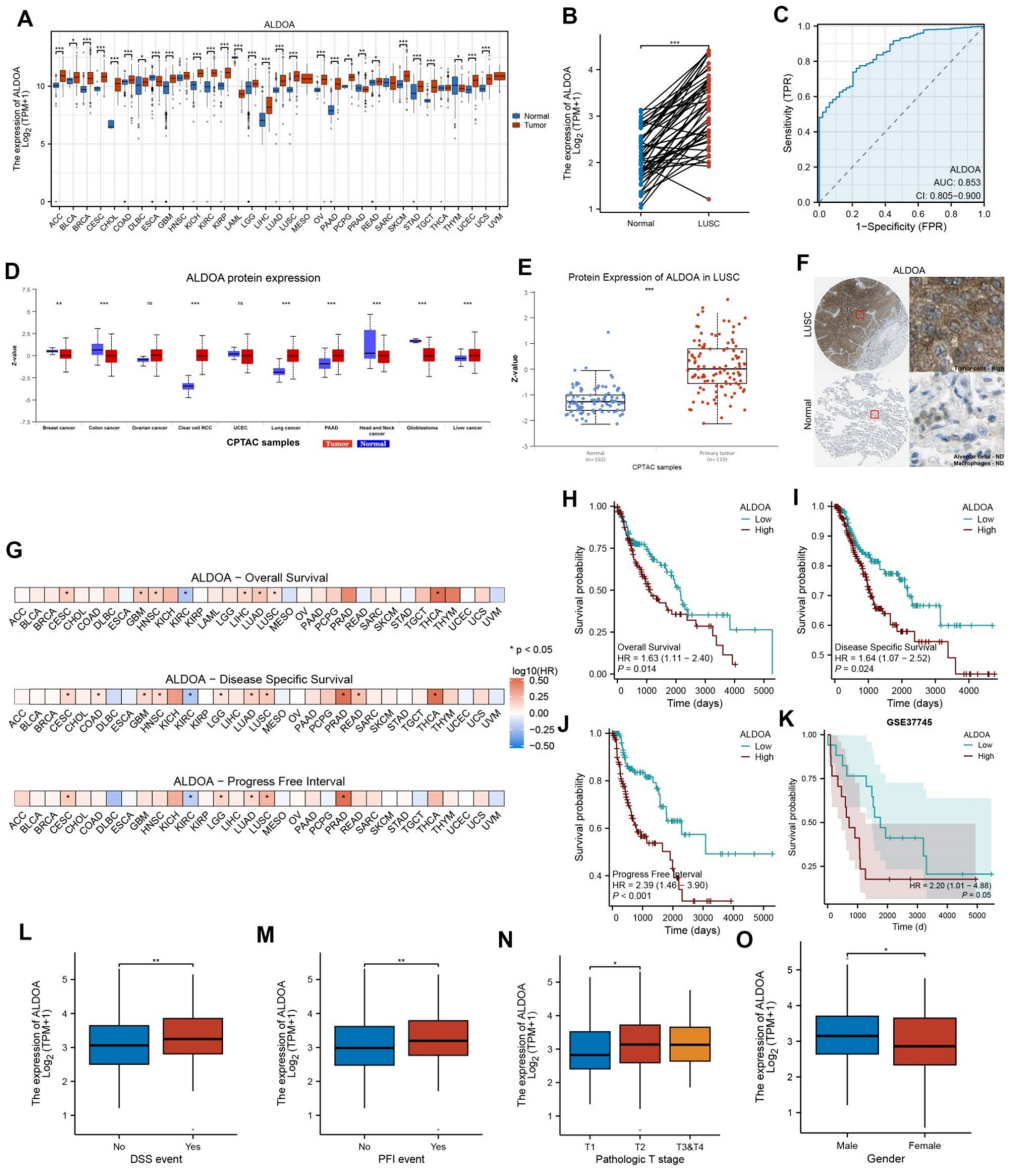
Expression and prognostic relevance of ALDOA in pan-cancer and LUSC. (**A**) Pan-cancer mRNA expression levels of ALDOA in tumor versus normal tissues across TCGA cohorts. (**B**) Paired comparison of ALDOA expression between LUSC tumor and adjacent normal tissues. (**C**) ROC curve evaluating the diagnostic performance of ALDOA in distinguishing LUSC from normal tissue. (**D**) Protein expression of ALDOA across cancers based on CPTAC proteomic data. (**E**) ALDOA protein expression levels in LUSC tumor and normal tissues from the CPTAC dataset. (**F**) Immunohistochemical staining of ALDOA in LUSC and adjacent normal tissues obtained from the Human Protein Atlas. (**G**) Heatmap showing hazard ratios of ALDOA expression for overall survival (OS), disease-specific survival (DSS), and progression-free interval (PFI) across TCGA cancer types. (**H**-**J**) Kaplan-Meier survival analyses of ALDOA expression in the TCGA-LUSC cohort for OS (H), DSS (I), and PFI (J). (**K**) Kaplan-Meier survival analysis for overall survival in the GSE37745 LUSC dataset. (**L**-**O**) Boxplots showing ALDOA expression stratified by clinical parameters including DSS event (**L**), PFI event (**M**), pathologic T stage (**N**), and gender (**O**)

The mRNA expression levels of ALDOA are significantly higher various cancer types other than ESCA, PRAD and THYM, as assessed using data from TCGA (Fig. 1A). Protein expression analysis of ALDOA, using CPTAC datasets, also shows significantly elevated levels in multiple cancer types (Fig. 1D). ALDOA expression was significantly elevated in LUSC tumor tissues compared to normal tissues at both mRNA and protein levels (Fig. 1B and E, *p* < 0.001). Immunohistochemistry further demonstrated stronger staining of ALDOA in tumor tissues (Fig. 1F). ROC analysis revealed high diagnostic accuracy for distinguishing LUSC from normal tissues (AUC = 0.853, Fig. 1C). Survival heatmaps illustrate the hazard ratios (HR) of ALDOA expression for overall survival (OS), disease-specific survival (DSS), and progression-free interval (PFI) across various cancer types (Fig. 1G). In LUSC patients, high ALDOA expression was associated with significantly worse overall survival (OS; HR = 1.63, *p* = 0.014), disease-specific survival (DSS; HR = 1.64, *p* = 0.024), and progression-free survival (PFS; HR = 2.39, *p* < 0.001) (Fig. 1H-J). We validated these prognostic associations in an independent cohort (GSE37745 dataset, Fig. 1K). Additionally, a statistically significant difference was observed between T1 and T2 stages (*p* < 0.05), but no significant differences were found between other stage comparisons (Fig. 1N, *p* < 0.05). ALDOA expression was higher in patients with DSS and PFI events (Fig. 1L–M, *p* < 0.01) and male patients (Fig. 1O). These results highlight ALDOA as a potential diagnostic and prognostic biomarker in LUSC.

### Genomic alterations and instability landscape of ALDOA across cancers

**Fig. 2:**
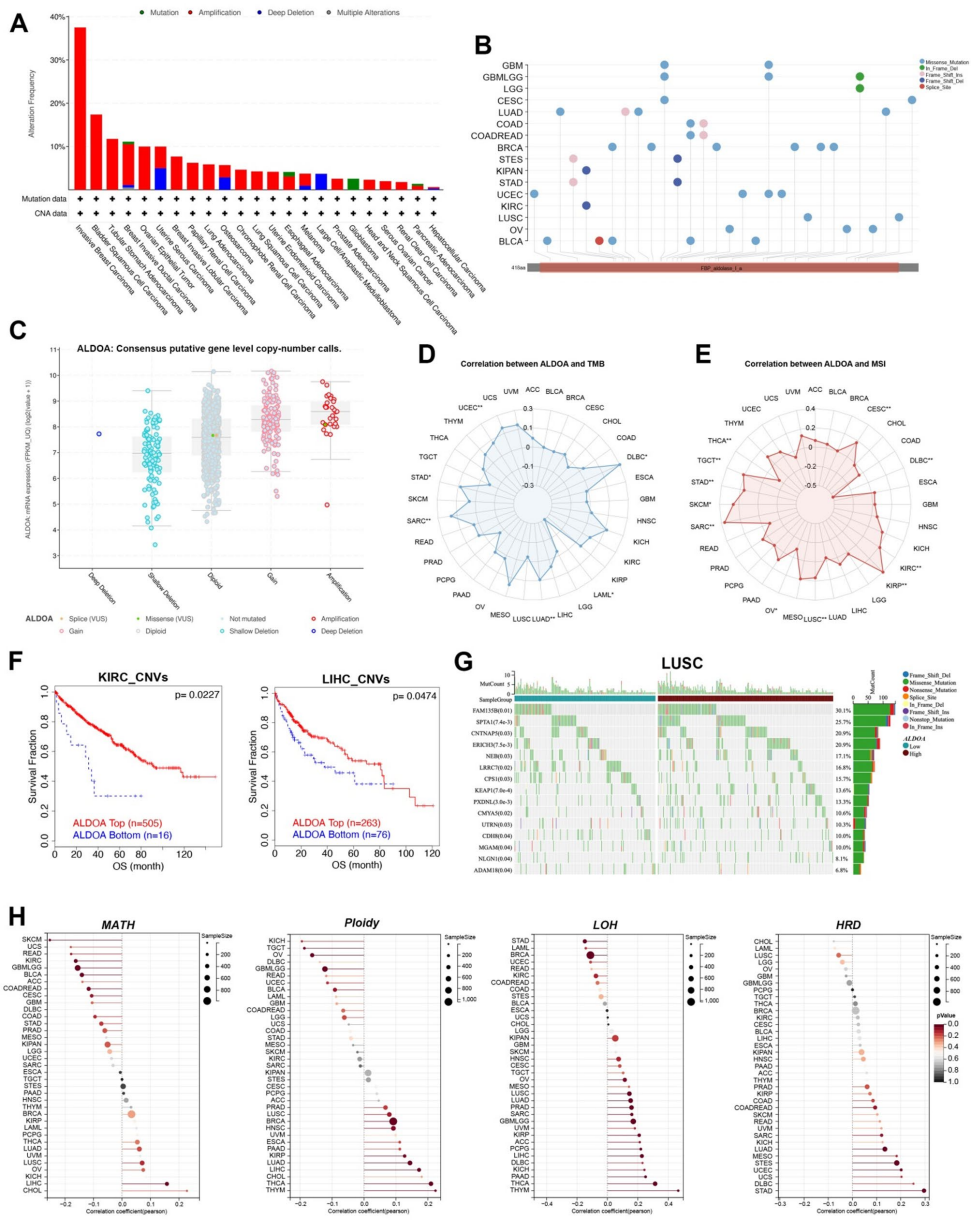
Genomic alterations and genomic instability landscape of ALDOA in pan-cancer and LUSC. (**A**) Frequency and types of ALDOA alterations across TCGA cancer types. (**B**) Distribution and classification of ALDOA mutations across cancer types, annotated by mutation type and domain location. (**C**) ALDOA mRNA expression levels in relation to gene-level CNV categories across pan-cancer samples. (**D**-**E**) Radar plots showing correlations between ALDOA expression and tumor mutational burden (TMB) (**D**) and microsatellite instability (MSI) (**E**), respectively, in different cancers. (**F**) Kaplan-Meier survival curves comparing overall survival between high and low ALDOA CNV groups in KIRC and LIHC cohorts. (**G**) Waterfall plot showing mutation profiles in LUSC patients stratified by ALDOA expression level. (**H**) Correlation between ALDOA expression and genomic instability markers, including mutant-allele tumor heterogeneity (MATH), ploidy, loss of heterozygosity (LOH), and homologous recombination deficiency (HRD), across cancer types

ALDOA alterations are most frequent in invasive breast carcinoma (nearly 40%), primarily driven by amplifications, with significant frequencies also observed in bladder cancer, uterine tumors, and ovarian epithelial tumors. LUSC indicates a moderate alteration frequency, primarily involving amplifications and some mutations (Fig. 2A). Missense mutations dominate across cancers, accompanied by frame-shift insertions, deletions, in-frame deletions, and splice-site mutations, with LUSC showing moderate alteration frequency, mainly amplifications and missense mutations within the FBP_aldolase_1_a domain, potentially affecting its functional region (Fig. 2B). CNV analysis shows that a trend of increasing ALDOA mRNA expression from “Deep Deletion” to “Amplification,” with the highest expression levels observed in the “Amplification” category. (Fig. 2C). Kaplan-Meier survival analysis highlights that higher ALDOA CNV levels are associated with worse overall survival in KIRC and LIHC (Fig. 2F, *p* < 0.05). Regarding tumor mutational burden (TMB), ALDOA expression showed a positive correlation in UCEC (*p* < 0.01) and STAD (*p* < 0.05) but a negative correlation in SARC (*p* < 0.01) and LAML (*p* < 0.05) (Fig. 2D). Similarly, ALDOA expression was positively correlated with microsatellite instability (MSI) in CESC (*p* < 0.01) and DLBC (*p* < 0.01) but negatively correlated in KIRC (*p* < 0.01) and LUSC (*p* < 0.01) (Fig. 2E), suggesting its potential role in maintaining genomic stability in LUSC.

Mutation profiling of LUSC samples stratified by ALDOA expression levels revealed distinct mutation landscapes (Fig. 2G). The most frequently mutated genes included FAM135B (30.1%), SPTA1 (25.7%) and CNTNAP5 (20.9%). Missense mutations were the dominant alteration type, followed by nonsense mutations, splice-site mutations, and frameshift insertions/deletions. These findings indicate that ALDOA-high LUSC cases exhibit a distinct mutational profile that may contribute to tumor progression. Additionally, ALDOA expression correlates with genomic instability markers, including MATH, ploidy, LOH, and HRD, suggesting its involvement in tumor heterogeneity and genomic instability across multiple cancers (Fig. 2H).

### Promoter methylation and epigenetic regulation of ALDOA in LUSC and pan-cancer

**Fig. 3:**
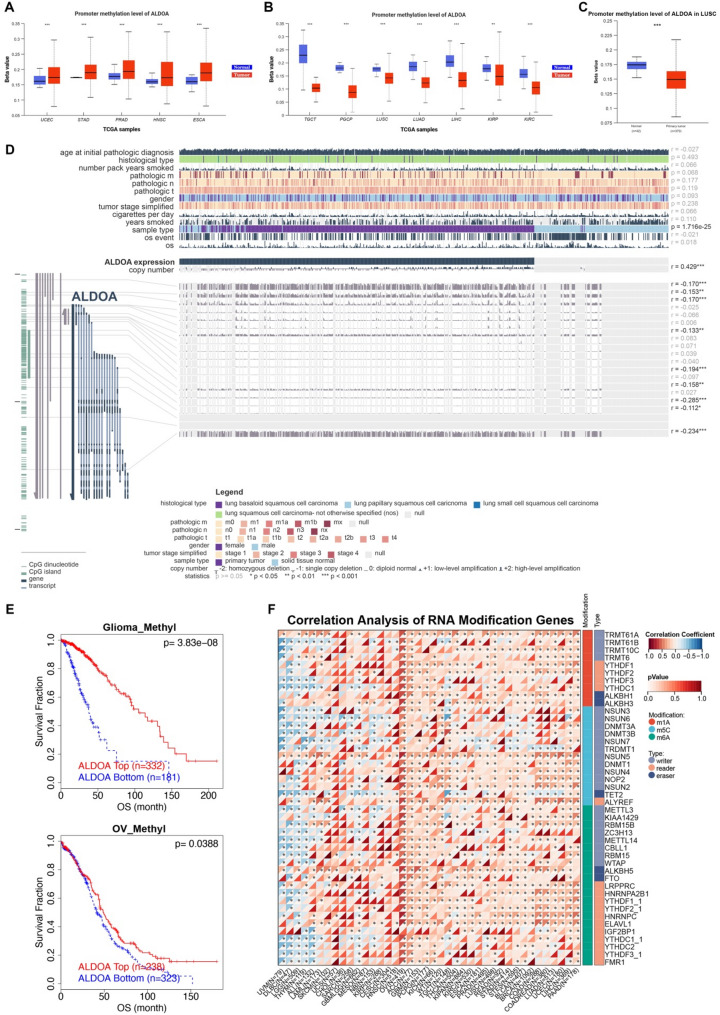
Promoter methylation and RNA modification-related regulation of ALDOA in pan-cancer and LUSC.(**A**-**B**) Boxplots showing promoter methylation levels of ALDOA in tumor versus normal tissues across TCGA cancer types. (**C**) Comparison of ALDOA promoter methylation between primary tumor and adjacent normal tissues in LUSC. (**D**) MEXPRESS visualization of ALDOA expression and its correlation with promoter CpG methylation, copy number variation, and clinical features in LUSC. (**E**) Kaplan-Meier survival curves comparing overall survival between high and low ALDOA methylation groups in glioma and ovarian cancer cohorts. (**F**) Correlation heatmap between ALDOA expression and genes related to RNA methylation modifications, including m1A, m5C, and m6A regulatory factors

Using the UCLAN database, we obtained the promoter methylation levels of ALDOA across various tumors, showing higher levels in some cancers (Fig. 3A) and lower levels in others (Fig. 3B). In LUSC, the promoter methylation levels of ALDOA are significantly reduced in primary tumors compared to normal tissues (*p* < 0.001) (Fig. 3C). MEXPRESS analysis revealed that ALDOA expression in LUSC positively correlated with copy number amplification (*r* = 0.429, *p* < 0.001) and negatively correlated with promoter methylation at multiple CpG sites. These findings suggest that copy number gains and promoter hypomethylation are key drivers of ALDOA overexpression, likely by enhancing its transcriptional activity. This regulatory mechanism may influence ALDOA’s role in LUSC progression and its utility as a biomarker. Survival analysis shows that hypomethylation of ALDOA in glioma and ovarian cancer is associated with significantly worse overall survival (*p* < 0.001 in glioma, *p* = 0.0388 in ovarian cancer) (Fig. 3E). Moreover, correlation analysis between ALDOA and RNA modification genes revealed strong associations with several RNA methylation regulators, including DNMT3A, METTL3, and ALKBH5, suggesting that ALDOA may be subject to additional post-transcriptional regulatory mechanisms (Fig. 3F).

### Aberrant alternative splicing of ALDOA is associated with tumor progression and poor prognosis

**Fig. 4:**
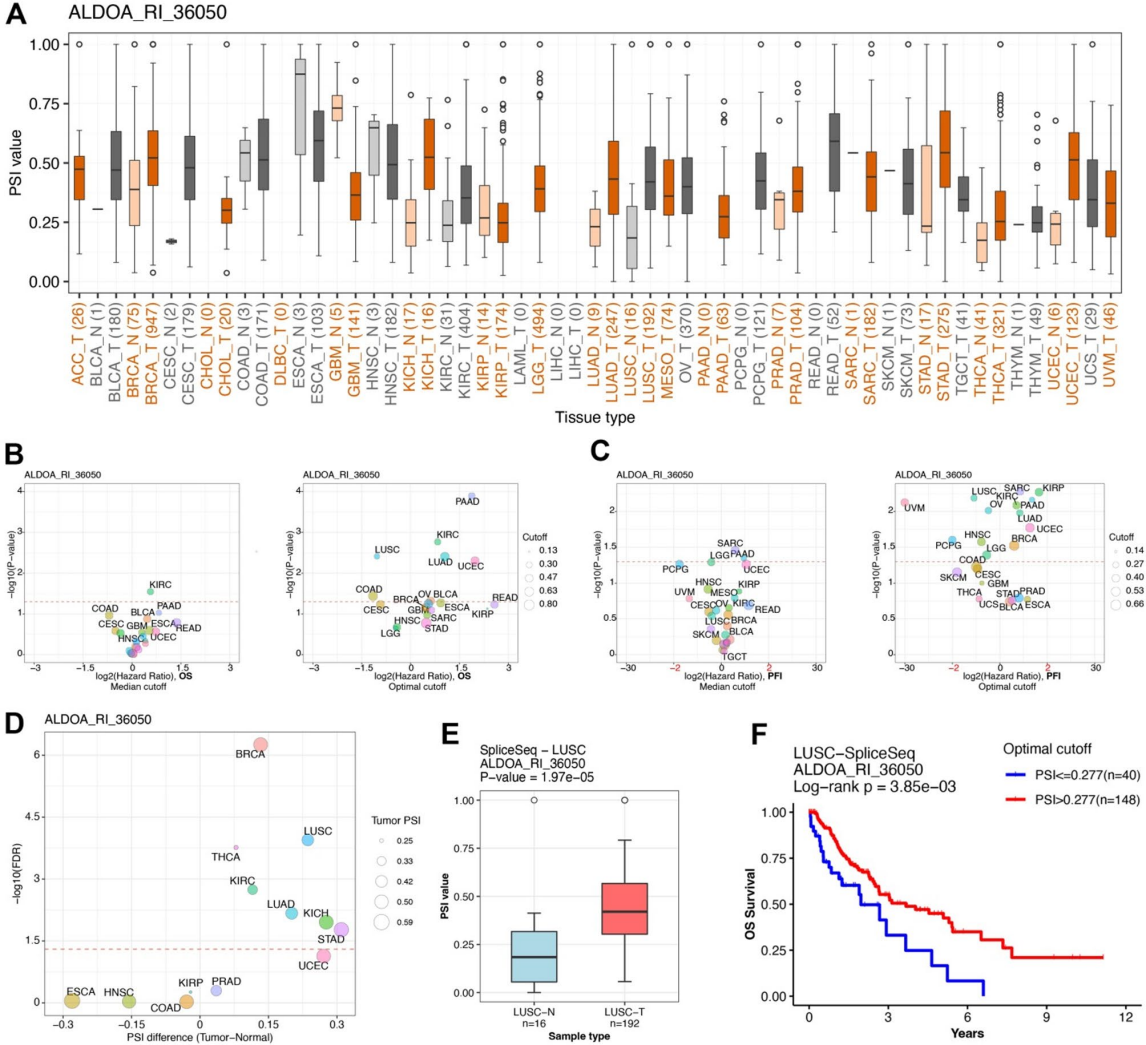
Tumor-specific alternative splicing event ALDOA_RI_36050 and its prognostic significance across cancers. (**A**) Boxplots of percent spliced-in (PSI) values of the ALDOA_RI_36050 event across tumor and normal tissues in multiple cancer types. (**B**-**C**) Volcano plots showing associations between ALDOA_RI_36050 PSI values and overall survival (OS) or progression-free interval (PFI) based on median and optimal cutoffs. (**D**) Differential PSI values of ALDOA_RI_36050 between tumor and normal samples across cancer types. (**E**) Comparison of ALDOA_RI_36050 PSI values between LUSC tumors and normal tissues in the SpliceSeq dataset. (**F**) Kaplan-Meier survival curve comparing OS in LUSC patients stratified by ALDOA_RI_36050 PSI using the optimal cutoff

Alternative splicing plays a critical role in tumorigenesis and cancer progression, influencing gene expression, protein diversity, and therapeutic responses [27–29]. Through the Oncosplicing database, we identified 130 ALDOA alternative splicing events, including ALDOA_RI_36050, which exhibited significant tumor-specific splicing pattern. In several tumor tissues, the PSI values exhibited a notable shift compared to their corresponding normal tissues, suggesting tumor-specific splicing alterations (Fig. 4A, D). Statistical analysis indicated that ALDOA_RI_36050 showed significantly differential splicing patterns in multiple cancer types, with Stomach Adenocarcinoma (STAD), Lung Squamous Cell Carcinoma (LUSC), and Kidney Chromophobe (KICH) displaying the most prominent differences.

To assess the clinical relevance of ALDOA_RI_36050, we performed survival analysis using median and optimal PSI cutoffs. For overall survival (OS), the median cutoff identified a significant association in KIRC, while the optimal cutoff extended this correlation to LUSC and UCEC (Fig. 4B). Regarding progression-free interval (PFI), the median cutoff linked ALDOA_RI_36050 splicing to disease progression in SARC and PAAD, with the optimal cutoff further highlighting its prognostic value in LUSC and KIRP (Fig. 4C). Focusing on LUSC, tumor samples showed a significantly higher PSI compared to adjacent normal tissues (*P* = 1.97 × 10⁻⁵, Fig. 4E). Kaplan-Meier survival analysis further confirmed that LUSC patients with higher PSI values (PSI > 0.277) had significantly poorer overall survival compared to those with lower PSI values (PSI ≤ 0.277) (log-rank *P* = 3.85 × 10⁻³, Fig. 4F). These findings suggest that the ALDOA_RI_36050 splicing event may serve as a potential prognostic biomarker in LUSC and other cancer types.

### Functional pathway enrichment and drug sensitivity of ALDOA in LUSC

**Fig. 5:**
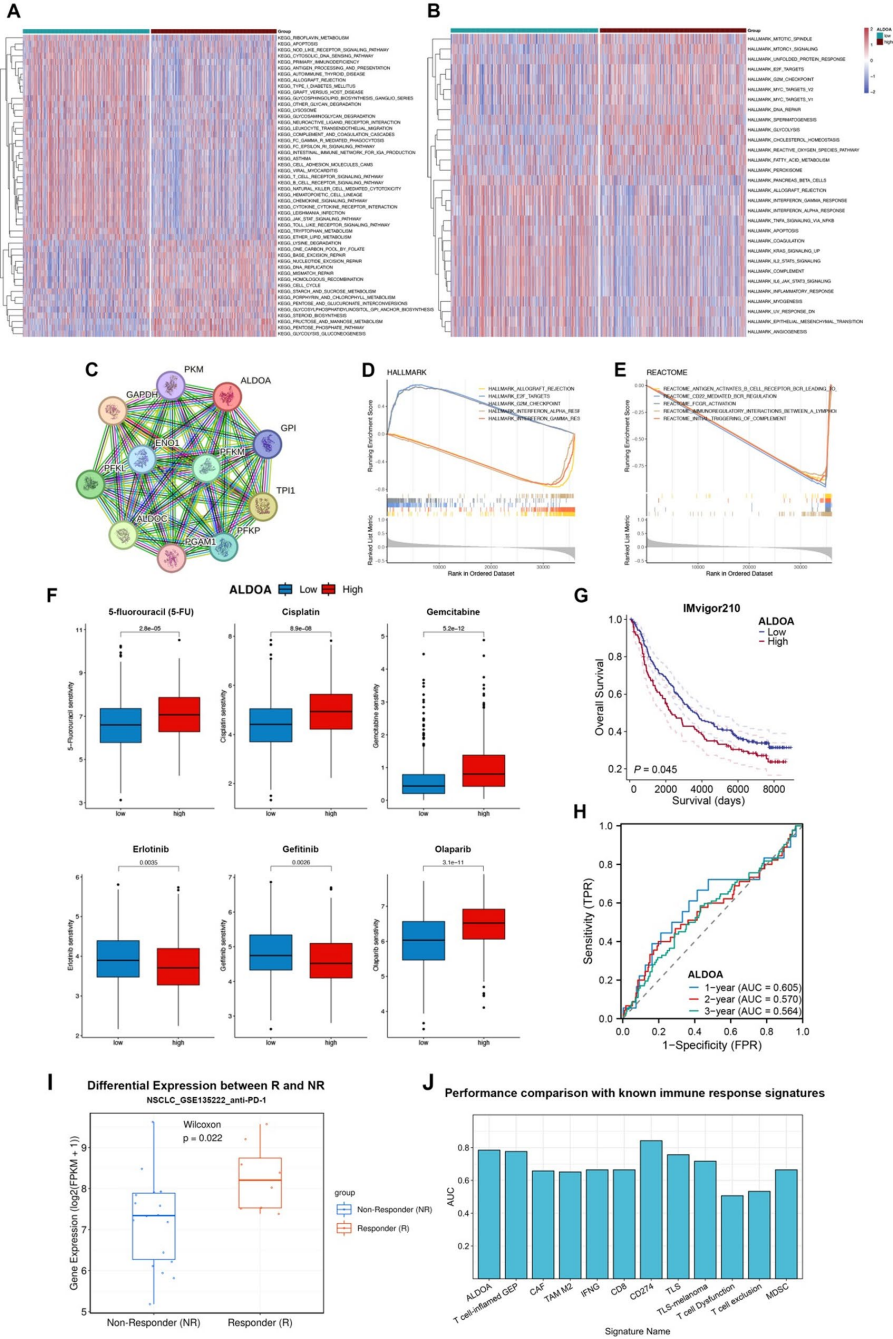
Functional pathway enrichment and drug sensitivity analysis associated with ALDOA expression in LUSC. (**A**-**B**) Heatmaps of KEGG (A) and HALLMARK (B) pathway enrichment scores based on Gene Set Variation Analysis (GSVA) comparing ALDOA-high and ALDOA-low groups. (**C**) Protein-protein interaction (PPI) network of ALDOA and its interacting partners derived from STRING database. (**D**-**E**) Gene Set Enrichment Analysis (GSEA) showing Hallmark (**D**) and Reactome (**E**) pathways enriched in the ALDOA-high group. (**F**) Boxplots comparing drug sensitivity (estimated IC50 values) between ALDOA-high and ALDOA-low groups for commonly used chemotherapeutic and targeted agents. (**G**) Kaplan-Meier survival analysis in the IMvigor210 immunotherapy cohort stratified by ALDOA expression. (**H**) ROC curves evaluating ALDOA’s predictive accuracy for 1-, 2-, and 3-year survival outcomes following immunotherapy. (**I**) Boxplot showing ALDOA expression in Responder versus Non-Responder groups from the NSCLC_GSE135222 dataset. (**J**) Bar plot comparing the AUC values of ALDOA with known immune response signatures (including T cell-inflamed GEP, CAF, TAM M2, IFNG, CD8, CD274, TLS, and TLS-melanoma) for predicting immunotherapy response

We further performed Gene Set Variation Analysis (GSVA) to compare KEGG and HALLMARK pathways between ALDOA-high and ALDOA-low groups utilizing TCGA-LUSC cohort (Table S1). The heatmaps demonstrate significant pathway differences, with high-ALDOA group associated with cell cycle progression, metabolic processes, and oncogenic signaling and low-ALDOA group associated with immune-related pathway, inflammation and apoptosis (Fig. 5A, B). Protein-protein interaction (PPI) network analysis identified strong interactions between ALDOA and key glycolytic enzymes (GAPDH, PKM, TPI1), as well as cytoskeletal proteins (ACTG1) and stress-response regulators (HSP90AA1), indicating its involvement in both metabolic and structural regulation (Fig. 5C). Furthermore, GSEA analysis confirmed that high ALDOA expression correlates positively with Hallmark cell cycle pathways and negatively correlates with inflammation-related pathway as well as immune-related signaling pathways in the Reactome database (Fig. 5D, E). These findings indicate that ALDOA functions as a key metabolic and oncogenic driver in LUSC, making it a potential therapeutic target.

Additionally, drug sensitivity analysis indicated that ALDOA expression levels were significantly correlated with IC50 values of multiple chemotherapy and targeted agents (Fig. 5F, Table S2). The ALDOA-low group exhibited increased sensitivity to EGFR-TKIs such as erlotinib and gefitinib, suggesting that ALDOA expression may be associated with resistance to EGFR-targeted therapies. Conversely, the ALDOA-high group showed higher resistance to 5-fluorouracil (5-FU), cisplatin, gemcitabine, and the PARP inhibitor Olaparib, indicating a potential role in modulating DNA damage repair pathways. These findings suggest that ALDOA may serve as a predictive biomarker for both EGFR-TKI efficacy and chemoresistance in LUSC.

In terms of immunotherapy response, analysis of the IMvigor210 cohort demonstrated that ALDOA-high patients had significantly worse survival outcomes following anti-PD-L1 therapy (*p* = 0.045) (Fig. 5G). Receiver operating characteristic (ROC) analysis further suggested that ALDOA could be a potential predictor for short-term immunotherapy response (AUC = 0.605 at 1 year, Fig. 5H). To further validate ALDOA’s role in predicting immunotherapy efficacy, we analyzed the NSCLC_GSE135222 dataset. Strikingly, ALDOA expression was significantly elevated in the Responder Group (patients with complete or partial response, CR/PR) compared to the Non-Responder Group (patients with stable or progressive disease, SD/PD) (Fig. 5I). Performance comparison with known immune response signatures revealed that ALDOA alone achieved an AUC of 0.7847 for predicting immunotherapy response, which was comparable to or even superior than most established multi-gene signatures, including T cell-inflamed GEP (AUC = 0.7763), CAF (AUC = 0.6579), TAM M2 (AUC = 0.6513), IFNG (AUC = 0.6645), CD8 (AUC = 0.6645), CD274 (AUC = 0.8421), TLS (AUC = 0.7566), and TLS-melanoma (AUC = 0.7171) (Fig. 5J). These results highlight ALDOA’s potential role in regulating immune responses and its possible implications as a biomarker for immunotherapy resistance in LUSC.

### ALDOA expression correlates with immune landscape and immune evasion in LUSC

**Fig. 6:**
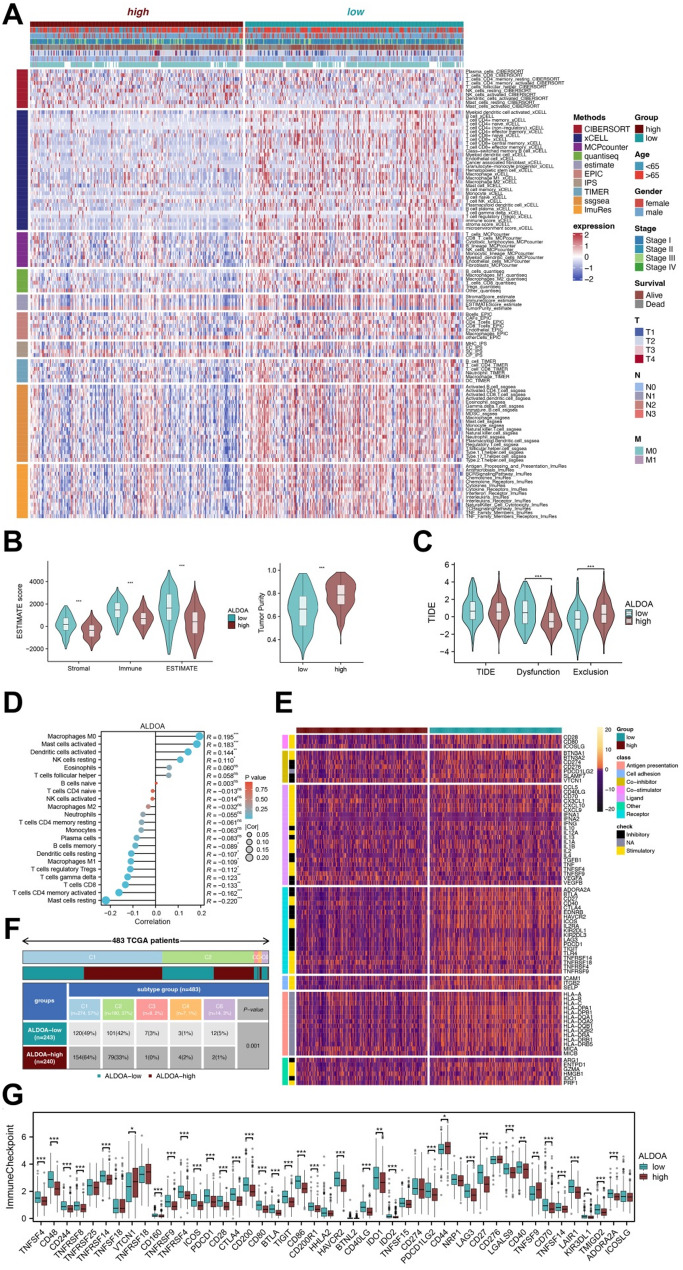
Immune landscape and immunosuppressive features associated with ALDOA expression in LUSC. (**A**) Heatmap of immune cell infiltration scores estimated by multiple algorithms across ALDOA-high and ALDOA-low groups in the TCGA-LUSC cohort. (**B**) Violin plots showing differences in stromal score, immune score, ESTIMATE score, and tumor purity between ALDOA expression groups. (**C**) Correlation analysis between ALDOA expression and the abundance of various immune cell types. (**D**) Distribution of immune subtypes (C1-C6) among ALDOA-high and ALDOA-low LUSC patients. (**E**) Heatmap of immune-related gene expression stratified by ALDOA expression status. (**F**) Violin plots of TIDE, immune dysfunction, and immune exclusion scores comparing ALDOA-high and ALDOA-low groups. (**G**) Boxplots comparing expression of classical immune checkpoint genes between ALDOA-high and ALDOA-low tumors

To further explore the role of ALDOA in the tumor immune microenvironment (TIME) of LUSC, we conducted a series of immune infiltration and immune checkpoint analyses. Utilizing multiple immune deconvolution algorithms, we assessed the relationship between ALDOA expression and immune cell composition. The analysis revealed significant immune landscape differences between the ALDOA-high and ALDOA-low groups (Fig. 6A, Table S3). Most immune cells, including CD8 + T cells and NK cells, were enriched in the ALDOA-low group, whereas the ALDOA-high group exhibited a more immunosuppressive TIME. ESTIMATE analysis further confirmed that the ALDOA-high group exhibited significantly lower stromal and immune ESTIMATE scores, but higher tumor purity scores compared to the ALDOA-low group (Fig. 6B). These findings suggest that ALDOA overexpression is associated with a less immunologically active TIME, which may contribute to tumor immune escape.

Next, we investigated the correlation between ALDOA expression and immune cell abundance (Fig. 6D). Notably, ALDOA expression was most strongly correlated with the M0 macrophage subset, implying that ALDOA may be involved in macrophage polarization and tumor-associated macrophage (TAM)-mediated immunosuppression. Immune subtype analysis (Fig. 6F) further revealed that ALDOA-high tumors were predominantly associated with the C1 (Wound Healing) subtype, indicating an angiogenesis and oncogenic proliferating role for ALDOA in certain tumor contexts. In contrast, ALDOA-low tumors were enriched in the C2 (IFN-γ-dominant) subtype, which is typically associated with enhanced anti-tumor immunity.

In terms of immune regulation, ALDOA-high tumors exhibited differential expression of immune-modulatory genes (Fig. 6E), suggesting that ALDOA might contribute to immune evasion mechanisms through the modulation of immune checkpoints and regulatory pathways. Analysis of TIDE (Tumor Immune Dysfunction and Exclusion) scores (Fig. 6C, Table S4) showed that the ALDOA-high group had significantly higher Exclusion scores and lower Dysfunction scores, supporting the idea that ALDOA overexpression may facilitate immune exclusion, while lessening immune cell dysfunction in the tumor microenvironment.

Finally, we compared the expression of classical immune checkpoint genes between the ALDOA-high and ALDOA-low groups (Fig. 6G, Table S5). Interestingly, ALDOA-high tumors exhibited lower expression of most immune checkpoint genes, including PDCD1 (PD-1), CTLA4, TIGIT and LAG3, suggesting that ALDOA overexpression may reduce immune checkpoint activity, potentially promoting immune evasion by decreasing immune cell engagement with the tumor.

Taken together, these findings indicate that ALDOA plays a crucial role in shaping the immune landscape of LUSC, with its overexpression associated with immune evasion, macrophage infiltration, and the activation of immune checkpoint pathways. This suggests that ALDOA may represent a potential target for modulating the immune response in LUSC therapy.

### Single-cell and Spatial transcriptomics analysis of ALDOA expression in cancer and its association with macrophages

**Fig. 7:**
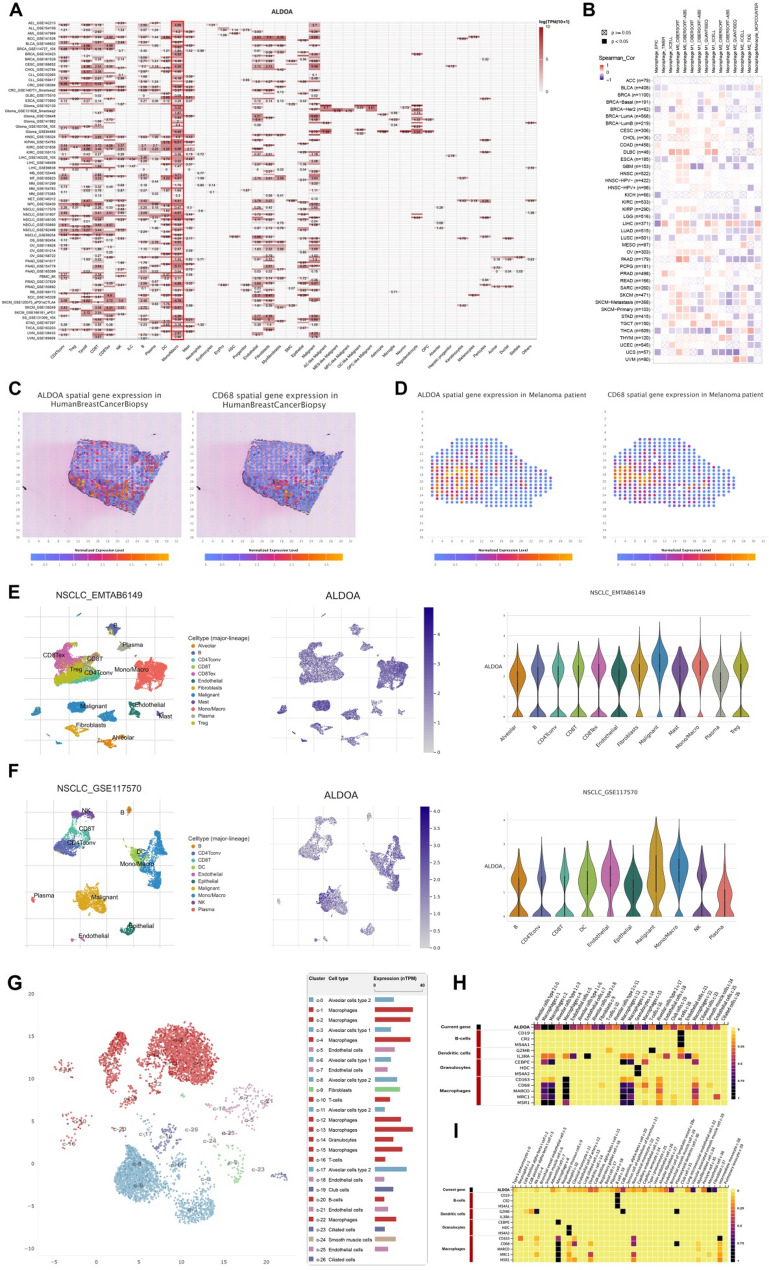
Single-cell and spatial transcriptomic analysis of ALDOA expression and its association with macrophages in multiple cancers. (**A**) Heatmap showing ALDOA expression across different cell types in pan-cancer single-cell RNA-seq datasets from the TISCH2 database. (**B**) Correlation matrix between ALDOA expression and macrophage across various cancers. (**C**-**D**) Spatial transcriptomic visualization of ALDOA and CD68 expression in breast cancer (**C**) and melanoma tissues (**D**), highlighting spatial co-expression patterns. (**E**-**F**) Single-cell expression profiles of ALDOA across major cell lineages in two NSCLC datasets (EMTAB6149 and GSE117570), shown as UMAP plots and violin plots. (**G**) Single-cell resolution analysis of ALDOA expression in normal human lung tissue from the Human Protein Atlas (HPA) database. Left: UMAP projection of all identified cell clusters, with ALDOA RNA expression overlaid. Right: Bar plot quantifying the proportion of ALDOA-expressing cells within each cluster, demonstrating predominant expression in macrophages. (**H**-**I**) Cell marker heatmaps from the HPA database, specifically from the “SINGLE CELL TYPE” (**H**) and “Tabula Sapiens” (**I**) projects, demonstrating strong co-expression of ALDOA with classical macrophage markers (CD163, CD68, MARCO, MRC1, MSR1) in normal lung tissue

To investigate the cell-type-specific expression of ALDOA across different cancer types, we performed single-cell RNA sequencing analysis using the TISCH2 database. The heatmap revealed that ALDOA is highly expressed in macrophages across multiple cancer types (Fig. 7A). This suggests a potential role for ALDOA in shaping the tumor immune microenvironment (TIME) by influencing macrophage infiltration and polarization. Subsequent correlation analysis between ALDOA expression and macrophage markers further validated a significant association between ALDOA and macrophage infiltration in different cancer types (Fig. 7B).

Spatial transcriptomic data of CD68, a macrophage marker, was used to confirm this relationship, showing co-expression of ALDOA and CD68 in macrophages within both breast cancer and melanoma samples (Fig. 7C). Additionally, the expression of ALDOA was explored in two distinct NSCLC (Non-Small Cell Lung Cancer) single-cell datasets (Fig. 7E and F). Together, these findings highlight ALDOA’s dual function in both tumor cells and immune cells, particularly tumor-associated macrophages, and its potential impact on macrophage-driven tumor biology, suggesting that ALDOA may contribute to macrophage-mediated immune suppression and tumor progression in LUSC.

To unequivocally determine the cellular origin of ALDOA within the pulmonary microenvironment and validate its specific enrichment in macrophages, we interrogated the Human Protein Atlas (HPA) database. Single-cell RNA-seq data from normal human lung tissue demonstrated that ALDOA expression was predominantly localized to macrophages. The UMAP visualization and its corresponding bar plot clearly illustrated a high proportion of ALDOA-expressing cells within the macrophage clusters (Fig. 7G). Furthermore, cell marker heatmaps from both the “SINGLE CELL TYPE” and “Tabula Sapiens” projects robustly confirmed the strong co-expression pattern between ALDOA and classical macrophage markers, including CD163, CD68, MARCO, MRC1, and MSR1 (Fig. 7H and I). These findings from normal lung tissue solidify the intrinsic link between ALDOA and macrophages, independent of the tumor context.

### Proteomic and histological validation of ALDOA expression in LUSC

**Fig. 8:**
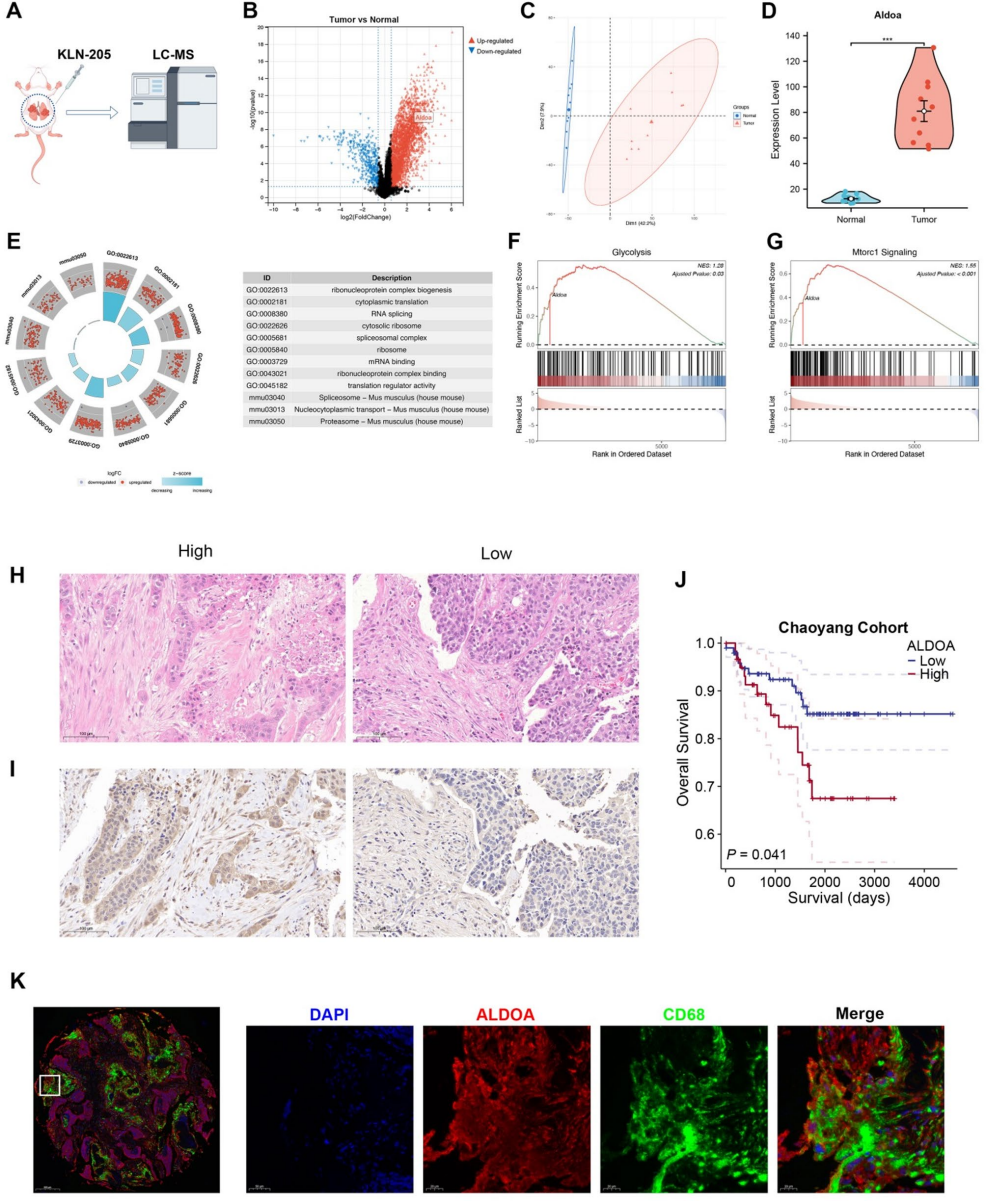
Proteomic and histological validation of ALDOA expression in LUSC using in vivo models and patient cohorts. (**A**) Schematic workflow of KLN-205 mouse orthotopic LUSC model and downstream proteomic analysis by LC-MS/MS. (**B**) Volcano plot showing differentially expressed proteins between tumor and adjacent normal tissues. (**C**) Principal component analysis (PCA) of proteomic profiles distinguishing tumor and normal samples. (**D**) Violin plot displaying Aldoa protein abundance between tumor and normal groups. (**E**) GO and KEGG pathway enrichment analyses of differentially expressed proteins based on MS data. (**F**–**G**) Gene Set Enrichment Analysis (GSEA) identifying glycolysis and mTORC1 signaling pathways enriched in Aldoa-high tumors. (**H**) HE-stained images for histological comparison of LUSC tumors. (**I**) Immunohistochemical staining of ALDOA in LUSC samples from the in-house cohort. (**J**) Kaplan-Meier survival analysis stratified by ALDOA expression in the Chaoyang in-house LUSC cohort. (**K**) Immunofluorescence images showing co-localization of ALDOA (red) and CD68 (green) in LUSC tissue, with DAPI nuclear counterstaining (blue) and merged image

To further investigate the role of ALDOA in LUSC^30^, a xenograft (PDX) model was established using the KLN-205 LUSC cell line, followed by proteomic analysis using liquid chromatography-mass spectrometry (LC-MS) (Fig. 8A, Table S6). Comparative proteomic analysis revealed a significant upregulation of ALDOA in tumor tissues compared to adjacent normal counterparts. Volcano plot analysis (Fig. 8B) identified Aldoa as a differentially upregulated protein, while principal component analysis (PCA) (Fig. 8C) confirmed a clear distinction between tumor and normal samples based on Aldoa expression. Quantitative protein expression analysis (Fig. 8D) further confirmed that Aldoa levels were significantly elevated in tumor samples.

GO and KEGG pathway enrichment analyses were performed on differentially expressed proteins (Fig. 8E), identifying significant enrichment in ribosome biogenesis and RNA processing. Additionally, GSEA analysis further supported the involvement of Aldoa in glycolysis and mTORC1 signaling pathways (Fig. 8F-G, Table S7), reinforcing its role in energy metabolism and oncogenic signaling.

Histological validation of ALDOA expression was conducted using hematoxylin-eosin (HE) staining and immunohistochemistry (IHC) (Fig. 8H-I). Tumor samples with higher ALDOA expression exhibited more pronounced histopathological alterations. Survival analysis of our In-house cohort revealed that patients with high ALDOA expression had significantly worse overall survival (OS) compared to those with low ALDOA expression (Fig. 8J, Table S8), highlighting its potential prognostic value. Furthermore, immunofluorescence (IF) staining demonstrated co-localization of ALDOA with CD68 + tumor-associated macrophages (TAMs) (Fig. 8K), suggesting a possible link between ALDOA expression and the immunosuppressive tumor microenvironment. These results indicate that ALDOA is significantly overexpressed in LUSC at the protein level, contributing to metabolic reprogramming, tumor progression, and poor clinical outcomes. Its association with tumor-associated macrophages suggests a potential role in shaping the immune microenvironment, further supporting its value as both a diagnostic and prognostic biomarker in LUSC.

### ALDOA expression is upregulated in tumors responsive to anti-PD-1 therapy

**Fig. 9:**
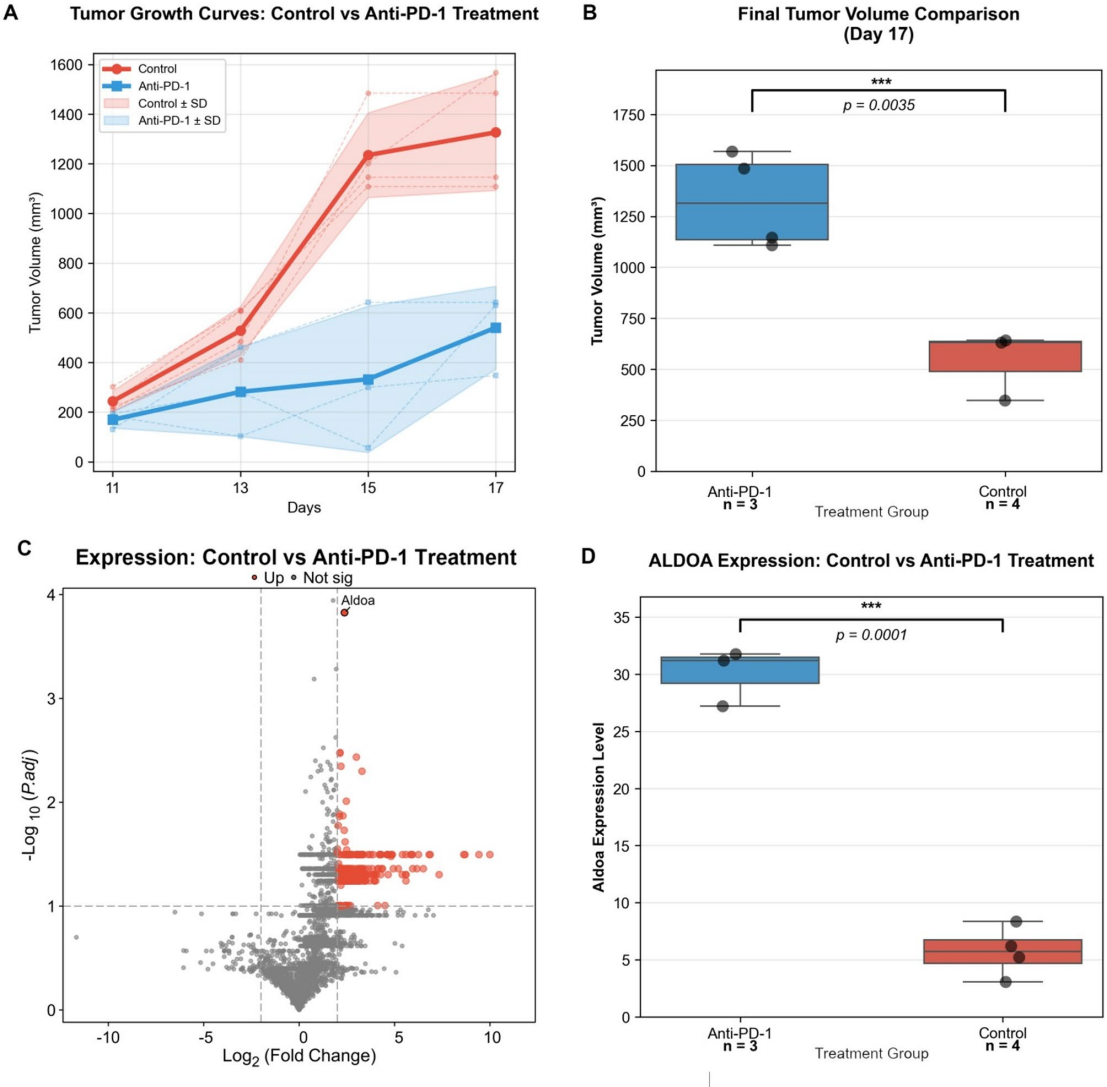
ALDOA expression is associated with response to anti-PD-1 therapy in a LUSC mouse model. (**A**) Tumor growth curves in KLN-205 allograft mice treated with anti-PD-1 or control antibody. Tumor volumes were showed at days 11, 13, 15, and 17 post-implantation. Data are presented as mean ± SD (*n* = 4 for Control group, *n* = 3 for Anti-PD-1 group). Individual tumor growth trajectories are shown as semi-transparent dotted lines. (**B**) Comparison of final tumor volumes at day 17 post-treatment. Box plots show the median (center line), interquartile range (box), and range (whiskers) of tumor volumes in control (*n* = 4) and anti-PD-1 treated (*n* = 3) groups. Individual data points are overlaid as black dots. Statistical significance was determined by unpaired two-tailed t-test (*p* = 0.0047). (**C**) Volcano plot depicting differentially expressed proteins between anti-PD-1-treated and control tumors from LC-MS/MS analysis. Aldoa is highlighted in red. (**D**) Box plot comparing Aldoa protein abundance in tumors from the two treatment groups

To experimentally validate the relationship between ALDOA and immunotherapy response, we treated KLN-205 allograft mice with anti-PD-1 antibody or an isotype control. Treatment with anti-PD-1 significantly suppressed tumor growth compared to the control group (Fig. 9A). This was further confirmed by a striking reduction in the final tumor volumes in the anti-PD-1-treated cohort (*p* < 0.001, Fig. 9B).Proteomic profiling by LC-MS/MS revealed a distinct protein expression pattern between the two groups (Fig. 9C). Notably, Aldoa was significantly upregulated in the anti-PD-1-treated tumors compared to controls (*p* < 0.01, Fig. 9D), suggesting that elevated Aldoa expression may be associated with a favorable response to anti-PD-1 therapy.

These in vivo findings further support the potential of ALDOA as a predictive biomarker for immunotherapy response in LUSC, and indicate that its upregulation may reflect a more immunogenic tumor microenvironment susceptible to checkpoint blockade.

## Discussion

Lung squamous cell carcinoma (LUSC) remains a major clinical challenge due to its high aggressiveness, poor prognosis, and limited treatment options [15, 30]. While significant progress has been made in identifying molecular drivers of LUSC [3, 28], novel biomarkers with diagnostic, prognostic, and therapeutic potential are urgently needed [31]. Recent studies highlight ALDOA (Aldolase A) as a crucial metabolic enzyme in cancer progression. It functions primarily through glycolysis and helps tumors adapt to metabolic stress [26]. For example, in hepatocellular carcinoma, targeting ALDOA has been shown to disrupt glycolytic balance and induce energy stress due to the accumulation of fructose-1,6-bisphosphate (FBP), thereby impairing tumor cell survival [5]. These findings emphasize the importance of ALDOA as a potential therapeutic target in glycolysis-dependent tumors. In addition to metabolic regulation, ALDOA has been implicated in immune modulation, with emerging evidence suggesting that its expression correlates with immune cell infiltration patterns in tumors such as hepatocellular carcinoma [3]. However, the precise role of ALDOA in LUSC, particularly in immune evasion, remains largely unexplored. Given the metabolic and immunological heterogeneity of tumors, a pan-cancer analysis of ALDOA is essential. This approach can clarify its oncogenic role across malignancies and identify tumor-specific regulatory mechanisms. Therefore, our study aims to systematically investigate ALDOA expression across cancers with a particular focus on its prognostic value and immune-related functions in LUSC.

Our pan-cancer analysis revealed that ALDOA is significantly upregulated across multiple cancer types, including lung squamous cell carcinoma, liver hepatocellular carcinoma, bladder cancer, and ovarian cancer. The high expression of ALDOA in these tumors suggests its role as a potential oncogene with metabolic regulatory functions. Importantly, ALDOA overexpression is associated with poor survival in multiple cancers, further highlighting its relevance as a prognostic biomarker [3]. From a genomic perspective, copy number variations and promoter hypomethylation are key contributors to ALDOA overexpression in multiple tumors. Our analysis also linked ALDOA expression to tumor mutational burden and microsatellite instability in several cancers. This suggests a potential role for ALDOA in genomic instability and tumor progression. These findings indicate that ALDOA may function as a universal oncogenic driver with a conserved role in metabolic adaptation and tumor microenvironment remodeling across cancer types.

Among all cancer types, LUSC demonstrated one of the highest levels of ALDOA expression, and its clinical significance was further validated through multiple datasets [31]. ALDOA overexpression in LUSC was significantly correlated with advanced tumor stage, disease progression, and poor overall survival. Kaplan-Meier survival analysis confirmed that high ALDOA expression is associated with shorter overall survival, disease-specific survival, and progression-free interval, emphasizing its prognostic value in LUSC patients [32, 33]. Further validation using immunohistochemistry in patient-derived samples confirmed that ALDOA protein levels are significantly elevated in LUSC tumor tissues compared to normal adjacent tissues. Also, our in-house cohort containing 162 LUSC patients further validated that higher ALDOA expression was associated with worse overall survival, reinforcing its potential as a diagnostic and prognostic biomarker. These results indicate that ALDOA could serve as a useful clinical indicator for LUSC progression and patient stratification.

Our multi-omics approach and validation across independent cohorts provide compelling evidence linking ALDOA to an immunosuppressive TIME, particularly through its strong correlation and spatial co-localization with CD68 + macrophages. However, these findings primarily demonstrate a correlation. Future functional studies are imperative to establish a direct immunomodulatory role for ALDOA and decipher its underlying mechanism. Specifically, ALDOA knockdown or knockout models in LUSC cell lines, coupled with macrophage co-culture systems, would be essential. These experiments would determine if tumor-intrinsic ALDOA expression directly drives macrophage recruitment and polarizes them toward an immunosuppressive M2-like phenotype. Furthermore, these models could be used to investigate the potential molecular mediators, such as secreted cytokines or metabolic by-products (e.g., lactate), through which ALDOA-overexpressing tumor cells communicate with and reprogram tumor-associated macrophages.

The functional significance of ALDOA in the immune context is further underscored by our in vivo findings. The upregulation of Aldoa in tumors responding to anti-PD-1 therapy (Fig. 9) suggests a complex, and perhaps therapy-induced, role in the tumor-immune interplay. A knockdown model would be critical to test if ALDOA is necessary for this observed response or if its elevation is merely a bystander effect of a remodeled, more immunogenic microenvironment. Conversely, the predominant expression of ALDOA in macrophages within normal lung tissue (Fig. 7G-I) raises the intriguing possibility of an immune-cell intrinsic function. It remains to be determined whether macrophage-specific ALDOA expression contributes to their pro-tumoral functions independently of tumor cell ALDOA. Addressing these questions through functional assays is a crucial next step. This will validate ALDOA not only as a prognostic biomarker but also as a bona fide therapeutic target. Inhibiting ALDOA could disrupt key tumor-macrophage crosstalk pathways in LUSC.

The ALDOA gene encodes a member of the class I fructose-bisphosphate aldolase family, which is widely expressed at variable levels in the cytoplasm of most cell types [34]. Our alternative splicing analysis uncovered a tumor-specific retained intron event, ALDOA_RI_36050. This isoform is significantly upregulated in LUSC and portends poor survival. The precise functional consequences of this isoform require further validation. However, its strong clinical association allows for informed mechanistic speculation. Retained introns often introduce a premature termination codon (PTC), potentially targeting the mRNA for nonsense-mediated decay (NMD) and thereby fine-tuning the overall ALDOA transcript pool. However, given our observations of elevated total ALDOA protein in tumors (Fig. 8), it is less likely that NMD is the dominant outcome. A more plausible hypothesis is that the ALDOA_RI_36050 isoform yields a truncated protein or one with novel peptide sequences, if translated. This aberrant isoform could exhibit neomorphic (gain-of-new-function) activity or act in a dominant-negative manner. ALDOA has a canonical role as a glycolytic enzyme, catalyzing the reversible conversion of fructose-1,6-bisphosphate. It also has a suggested non-canonical function as a scaffolding protein that interacts with the cytoskeleton (Fig. 5C). Therefore, the RI-36,050 isoform might competitively disrupt the active tetramer formation, impairing glycolytic flux. Alternatively, it could acquire an altered binding affinity that preferentially drives oncogenic signaling, consistent with its multifaceted roles. Intriguingly, the association of this specific isoform with poor prognosis aligns with our core finding that high ALDOA activity shapes an immunosuppressive microenvironment characterized by M0 macrophage infiltration and T-cell exclusion (Figs. 6 and 7). It is plausible that the ALDOA_RI_36050 isoform contributes to this immune-evasive phenotype. Furthermore, the complex role of ALDOA in immunotherapy response—where we observed high total ALDOA expression associated with resistance in one cohort (IMvigor210, Fig. 5G) but with sensitivity in another (NSCLC_GSE135222 and our mouse model, Figs. 5I and 9)—hints at a layer of regulation that might be isoform-specific. The balance between full-length ALDOA and its spliced variants could ultimately dictate tumor cell fate in response to immune pressure. Future studies focusing on characterizing this and other ALDOA isoforms at the protein level and delineating their distinct roles in LUSC pathogenesis and immune modulation are warranted.

Macrophages play a crucial role in the tumor microenvironment (TME) and have been widely recognized for their ability to promote cancer progression through various mechanisms [35]. Tumor-associated macrophages (TAMs), particularly those polarized toward the M2 phenotype, contribute to tumor growth, angiogenesis, immune suppression, and metastasis [36]. Recent findings indicate that TAMs contribute to cancer progression through diverse mechanisms, including immune suppression, metabolic reprogramming, and the secretion of tumor-promoting factors [36]. Understanding these pathways is essential for developing novel therapeutic strategies targeting macrophage-mediated tumor promotion, such as TAM depletion, macrophage reprogramming, or combination immunotherapies to enhance anti-tumor immune responses [37]. A striking finding of our study is the strong correlation between ALDOA expression and macrophage infiltration in LUSC. Using multiple immune deconvolution algorithms and single-cell RNA sequencing analysis, we demonstrated that ALDOA is highly expressed in both malignant tumor cells and monocyte-derived macrophages within the tumor microenvironment. Further analysis of the tumor immune landscape revealed that ALDOA-high tumors exhibit significantly lower immune infiltration, increased tumor purity, and a predominantly immunosuppressive microenvironment. Notably, ALDOA expression showed a strong positive correlation with M0 macrophage infiltration but a negative correlation with activated CD8 + T cells, suggesting that ALDOA may facilitate immune evasion by promoting immunosuppressive macrophage polarization. To further validate these findings, spatial transcriptomics and immunofluorescence staining demonstrated that ALDOA co-localizes with CD68 + macrophages in LUSC tumor tissues, confirming its potential role in macrophage recruitment and activation. These results indicate that ALDOA may serve as a key regulator of macrophage-mediated immune suppression, contributing to an immune-excluded phenotype in LUSC.

Having established a correlation between ALDOA overexpression and an immunosuppressive, macrophage-rich TME, we next explored its potential mechanisms. Our data suggest that ALDOA likely fosters an immune-suppressive niche through multiple, non-mutually exclusive pathways. First, the most straightforward mechanism is metabolite-driven immunosuppression. As a pivotal glycolytic enzyme, high ALDOA expression inevitably accelerates glycolytic flux in tumor cells, leading to massive lactate production and secretion. Lactate is no longer considered a mere waste product but a potent signaling molecule that can recruit monocytes and polarize them towards a pro-tumor, M2-like phenotype [38, 39]. Concurrently, an acidic, lactate-rich microenvironment directly impairs the cytotoxic function and viability of CD8 + T cells and NK cells [40–44], effectively crippling the primary anti-tumor immune response. Thus, the ALDOA-driven ‘Warburg effect’ not only fulfills the bioenergetic and biosynthetic demands of the tumor but also actively constructs an immune-hostile niche [45]. Second, beyond its cell-autonomous role in tumor cell metabolism, our single-cell and spatial transcriptomic analyses unveil a more direct and intriguing connection between ALDOA and macrophages. We discovered that ALDOA is not only highly expressed in tumor cells but is also remarkably enriched in CD68 + macrophages themselves (Fig. 7A, E, F). This finding was further solidified by analysis of normal human lung tissue from the HPA database, which demonstrated that ALDOA expression is a inherent feature of pulmonary macrophages, co-expressing with classical markers like CD163, CD68, and MARCO (Fig. 7G-I). This suggests a dual role for ALDOA: driving tumor cell intrinsic glycolysis, and simultaneously functioning within the TAM population. It is plausible that ALDOA-mediated glycolysis is essential for the metabolic fitness, survival, and pro-tumor functions of TAMs within the hypoxic TME. Alternatively, tumor-derived ALDOA could be secreted (a non-canonical function described for some glycolytic enzymes [46, 47]) and act as a damage-associated molecular pattern (DAMP) or cytokine to directly engage receptors on macrophages, reinforcing their immunosuppressive polarization. Third, the relationship between ALDOA and therapy response revealed a layer of fascinating complexity. While our bioinformatic analysis (TIDE score) and data from the IMvigor210 cohort (Fig. 5G) suggested that ALDOA-high tumors might be resistant to immunotherapy, our analysis of the NSCLC_GSE135222 dataset and, more importantly, our in vivo mouse model told a more nuanced story. We found that ALDOA expression was significantly higher in patients responding to anti-PD-1 therapy (Fig. 5I) and was strikingly upregulated in KLN-205 tumors that responded to anti-PD-1 treatment (Fig. 9C-D). This apparent paradox can be explained by a “metabolic immune feedback” model. ALDOA-high tumors may represent a subset with intense glycolytic activity and potentially high tumor antigenicity, making them initially more visible to the immune system. The powerful T-cell reinvigoration upon checkpoint blockade likely induces profound metabolic stress in tumor cells, triggering a compensatory upregulation of ALDOA and other glycolytic enzymes as a survival mechanism. Consequently, ALDOA upregulation in this context may be a biomarker of productive anti-tumor immunity but also a harbinger of an emerging metabolic checkpoint of resistance. Targeting ALDOA in combination with immunotherapy could preemptively disrupt this adaptive response and prevent relapse.

Our Tumor Immune Dysfunction and Exclusion analysis showed that ALDOA-high tumors exhibit higher immune exclusion scores, suggesting that patients with elevated ALDOA expression may be less responsive to immune checkpoint blockade therapy. Furthermore, immune checkpoint analysis revealed that ALDOA-high tumors exhibit reduced expression of key immune checkpoints, including PDCD1, CTLA4, and TIGIT, potentially reducing immune cell engagement with the tumor and further promoting immune evasion.

Given its multifaceted role in metabolic regulation, tumor progression, and immune suppression, ALDOA represents an attractive therapeutic target for LUSC [32, 33]. Our findings suggest that targeting ALDOA-mediated metabolic reprogramming could be an effective therapeutic strategy. ALDOA is a key enzyme in glycolysis, and its high expression in LUSC suggests a strong dependency on aerobic glycolysis [1]. Targeting ALDOA-mediated glycolysis using metabolic inhibitors such as 2-DG or glycolysis inhibitors may disrupt tumor cell metabolism and impair tumor progression [48]. Moreover, given the strong correlation between ALDOA and macrophage-driven immune suppression, inhibiting ALDOA may enhance anti-tumor immune responses by reducing immunosuppressive macrophage polarization and increasing CD8 + T-cell infiltration. Combining ALDOA inhibitors with immune checkpoint inhibitors, such as anti-PD-1/PD-L1 or anti-CTLA4, may offer a promising therapeutic approach to overcoming immune evasion in LUSC. Furthermore, our drug sensitivity analysis revealed that ALDOA-high tumors exhibit increased resistance to chemotherapy agents, including 5-fluorouracil, cisplatin, gemcitabine, and PARP inhibitors such as Olaparib. Inhibiting ALDOA may enhance the efficacy of standard chemotherapeutic regimens, particularly in ALDOA-high LUSC patients.

Our study reveals a seemingly paradoxical finding: ALDOA-high LUSC tumors, despite being immunosuppressive, exhibited lower expression of key immune checkpoint genes such as PDCD1 (PD-1), CTLA4, and TIGIT (Fig. 6G).This counterintuitive observation is consistent with an ‘immune-excluded’ phenotype [49]. We propose two non-mutually exclusive explanations for this phenomenon. First, the reduced checkpoint expression is likely a direct reflection of the profoundly depleted T-cell infiltration in the ALDOA-high tumor core. Our immune deconvolution and ESTIMATE [50] analyses consistently demonstrated that ALDOA-high tumors are characterized by significantly fewer CD8 + T cells and higher tumor purity (Fig. 6A-C). Immune checkpoint molecules are predominantly expressed on tumor-infiltrating lymphocytes (TILs) [51, 52]. Therefore, a paucity of TILs naturally leads to an overall lower level of checkpoint gene transcripts in bulk tumor RNA-seq data. This suggests that the primary mechanism of immune evasion in ALDOA-high tumors is not the functional exhaustion of T cells (which is typically associated with high checkpoint expression), but rather the physical exclusion of T cells from the tumor microenvironment, potentially driven by the macrophage-rich, suppressive niche. Second, and more intriguingly, our immunotherapy response data provide a dynamic and nuanced perspective on this relationship. While baseline ALDOA-high tumors are ‘cold’ and immune-excluded, they may possess the potential to be converted into ‘hot’ tumors upon effective intervention. In the NSCLC_GSE135222 cohort, patients who responded to immunotherapy (CR/PR) had significantly higher ALDOA expression than non-responders (SD/PD) (Fig. 5I). In our KLN-205 mouse model, tumors responding to anti-PD-1 therapy showed a significant upregulation of Aldoa protein compared to controls (Fig. 9C-D). These findings suggest that ALDOA-high tumors, while initially ‘cold’, might be more susceptible to immune reactivation. The successful T-cell recruitment and reinvigoration by checkpoint blockade could subsequently lead to an increase in checkpoint molecule expression as a feedback mechanism. Thus, the low baseline checkpoint levels we observed may represent a ‘pre-engaged’ state where the immune system has been largely excluded and disengaged. Upon breaking the barrier (e.g., with anti-PD-1), T cells infiltrate and become activated, leading to the observed high ALDOA expression in responders, which could now be associated with elevated checkpoint levels—a hypothesis that warrants future investigation with paired pre- and post-treatment samples. In conclusion, low immune checkpoint expression in ALDOA-high LUSC indicates an immune-excluded, T-cell-desert TME. This is likely orchestrated by ALDOA-associated macrophage infiltration. However, this state should not be equated with immunotherapeutic irreversibility. Instead, ALDOA may serve as a biomarker for a specific subset of immune-excluded tumors that, upon successful reversal of exclusion, can mount a potent anti-tumor response.

This study provides comprehensive evidence that ALDOA is a crucial oncogene in LUSC, driving tumor progression, immune evasion, and therapy resistance. Our findings establish ALDOA as a robust prognostic biomarker and a promising therapeutic target, particularly in metabolism-driven and macrophage-enriched tumors. Future studies should focus on exploring the mechanistic role of ALDOA in macrophage polarization and immune evasion, validating ALDOA as a therapeutic target using in vivo models, and developing ALDOA-targeted inhibitors or metabolic drugs to enhance immunotherapy responses in LUSC. Given the growing importance of tumor metabolism and immune regulation in cancer therapy, ALDOA emerges as a critical link between metabolic adaptation and immune suppression, opening new avenues for targeted therapy in LUSC.

Despite the comprehensive multi-omics and multi-cohort validation presented herein, our study is not without limitations. Most critically, the compelling correlations between ALDOA overexpression, macrophage infiltration, and immune suppression primarily suggest association rather than direct causation. The absence of functional experiments, such as ALDOA knockdown or knockout in LUSC cell lines coupled with macrophage co-culture systems, precludes a definitive conclusion that tumor-intrinsic ALDOA directly orchestrates macrophage recruitment and polarization. Consequently, the precise molecular mechanisms—whether mediated by metabolic by-products like lactate [53], secreted cytokines [54], or other signaling molecules—underpinning this crosstalk remain elusive and warrant rigorous investigation. Furthermore, while we identified a prognostically significant alternative splicing event (ALDOA_RI_36050), its functional impact on protein structure, enzymatic activity, and immune modulation requires experimental validation at the protein level. The translational potential of ALDOA as a biomarker and target, though strongly supported by our data, ultimately necessitates validation through prospective clinical trials. Future research must therefore prioritize: (1) Mechanistic studies employing genetic manipulation models to dissect ALDOA’s cell-autonomous and non-autonomous roles in shaping the immunosuppressive TME; (2) Preclinical evaluation of ALDOA-targeting strategies, either alone or in rational combination with chemotherapy or immunotherapy, to assess their efficacy in reversing immune suppression and overcoming therapy resistance; and (3) The initiation of biomarker-driven clinical trials to prospectively validate the utility of ALDOA for patient stratification and its potential as a therapeutic target in LUSC. Addressing these limitations will be crucial for translating our findings into tangible clinical benefits for LUSC patients.

Our findings firmly establish ALDOA as a robust prognostic biomarker and a compelling therapeutic target in LUSC, with significant implications for clinical translation. The consistent association between high ALDOA expression and poor survival across TCGA, GEO, and our in-house cohort underscores its utility for risk stratification. In practice, ALDOA expression, easily detectable via IHC, could be integrated into clinical decision-making to identify high-risk patients who may benefit from more aggressive or novel treatment strategies. Beyond prognosis, our data reveal ALDOA’s central role in driving tumor progression through metabolic reprogramming, macrophage-mediated immune suppression, and therapy resistance, positioning it as a multi-faceted therapeutic node [45, 55]. The dual role of ALDOA in tumor cell-intrinsic metabolism and the immune microenvironment provides a strong rationale for several targeted approaches. Direct inhibition of ALDOA, though requiring further drug development, could disrupt the glycolytic backbone of these tumors. More immediately, combining existing metabolic modulators with standard chemotherapy could exploit the metabolic vulnerability of ALDOA-high tumors to overcome chemoresistance, as suggested by our drug sensitivity analysis.

## Conclusions

This study comprehensively elucidates the oncogenic role and clinical relevance of Aldolase A (ALDOA) across multiple cancer types, with a particular focus on lung squamous cell carcinoma (LUSC). We demonstrate that ALDOA is significantly overexpressed in LUSC and is strongly associated with poor prognosis, including reduced overall survival, disease-specific survival, and progression-free intervals. Our in-house cohort was used to independently validate the prognostic significance of ALDOA expression in LUSC, confirming its association with poor overall survival at both the transcriptomic and protein levels. Mechanistically, ALDOA overexpression is driven by genomic amplifications and promoter hypomethylation and is accompanied by a distinct pattern of immune suppression. High ALDOA expression in LUSC is associated with enhanced oncogenic signaling and glycolytic activity, while contributing to an immunosuppressive tumor microenvironment characterized by reduced CD8⁺ T-cell infiltration, increased M0 macrophage presence, and diminished immune checkpoint gene expression. Spatial transcriptomics and immunofluorescence staining further confirm the co-localization of ALDOA with CD68⁺ tumor-associated macrophages, suggesting a direct role in macrophage-mediated immune modulation. Targeting ALDOA may therefore represent a promising strategy to enhance the efficacy of metabolic inhibitors, immunotherapies, and combination treatment regimens in LUSC.

## Supplementary Information

Supplementary Material 1.

## Data Availability

All data generated or analyzed during this study are included in this published article and its supplementary information files. The transcriptomic and clinical data of TCGA pan-cancer and LUSC samples were obtained via the UCSC Xena platform (https://xenabrowser.net/datapages/). Single-cell RNA-seq datasets were acquired from the TISCH2 databases. Spatial transcriptomic analyses were performed using STOmicsDB portal. Additional gene expression datasets used for validation, including GSE37745, are publicly available from the GEO (https://www.ncbi.nlm.nih.gov/geo/) portals. All code and materials supporting the conclusions of this article are available from the corresponding authors upon reasonable request.
